# Integrated molecular and phenotypic analysis of onion (*Allium cepa* L.) germplasm reveals limited correspondence between genetic structure and phenotypic traits

**DOI:** 10.3389/fpls.2026.1849494

**Published:** 2026-06-03

**Authors:** Lovro Sinkovič, Hourieh Tavakoli Hasanaklou, Nina Karče Poljanšek, Jelka Šuštar Vozlič, Barbara Pipan

**Affiliations:** Crop Science Department, Agricultural Institute of Slovenia, Ljubljana, Slovenia

**Keywords:** *Allium cepa*, cytoplasmic variation, germplasm characterization, phenotypic diversity, plant breeding, population structure, Selection Index

## Abstract

The relationship between genetic structure and phenotypic variation in onion (*Allium cepa* L.) germplasm remains poorly understood, especially in heterogeneous open-pollinated material. In this study, we examined this relationship in onion accessions from the Slovenian Plant Gene Bank (SRGB). We combined cytoplasmic markers, nuclear SSR and ILP markers, and phenotypic descriptors to evaluate the SRGB onion collection. A total of 243 genotypes were analyzed, including 240 individuals from 60 accessions and three reference cultivars. Cytoplasmic markers revealed limited polymorphism. Their profiles showed no clear correspondence with nuclear genetic clusters. In contrast, nuclear markers showed high diversity and significant population structure. Most nuclear variation occurred within accessions. Bayesian clustering and multivariate analyses consistently identified three nuclear genetic clusters. Phenotypic differences among these clusters were significant (PERMANOVA, R² = 0.274, p = 0.001), particularly for bulb size and morphology traits. However, genetic ancestry explained only a limited proportion of trait variation, with the strongest association observed for bulb diameter (R² = 0.207). Consistent with these results, Selection Index values varied widely among accessions, and high-ranking accessions were found across all genetic clusters. Together, these findings indicate that nuclear genetic structure reflects broad phenotypic tendencies, but it does not fully predict multi-trait breeding-oriented performance. The SRGB onion collection is therefore a structured germplasm resource with useful variation for future onion breeding. These results show that accession-level evaluation is needed when selecting gene bank material for onion breeding and hybrid development.

## 1 Introduction

Onion (*Allium cepa* L.) is widely cultivated and exhibits considerable variation in bulb morphology, composition, and environmental adaptation (Lyngkhoi et al., 2021). Germplasm collections are central to conserving this diversity and supporting breeding programs (Ochar and Kim, 2023). However, onion improvement remains challenging because the crop is predominantly outcrossing, highly heterozygous, and often maintained as heterogeneous open-pollinated populations (McCallum et al., 2008; Lyngkhoi et al., 2021). For this reason, *ex situ* germplasm collections are important for conserving allelic diversity and supporting breeding and adaptation (Ochar and Kim, 2023). Effective use of these collections requires reliable characterization at both molecular and phenotypic levels. Nuclear molecular markers, including SSR and gene-based markers, are commonly used to assess genetic diversity and population structure in onion (McCallum et al., 2008; Lyngkhoi et al., 2021; Jayaswall et al., 2019). However, it remains unclear to what extent molecularly inferred genetic structure corresponds to phenotypic differentiation. This issue is especially relevant in outcrossing species such as onion, where high within-population variability may weaken genotype–phenotype relationships. In addition, nuclear markers reflect only part of the underlying genetic variation. Distinct cytoplasmic types, commonly classified as normal (N) and male-sterile (S and T) cytoplasm, differ in their ability to induce pollen sterility and are widely used in onion breeding programs (Engelke et al., 2003; Havey and Kim, 2021). Cytoplasmic variation is therefore important because it is associated with cytoplasmic male sterility (CMS), which underpins hybrid seed production. Despite its relevance for breeding, cytoplasmic diversity is less frequently characterized than nuclear diversity in onion germplasm studies, and its relationship with nuclear genetic structure remains insufficiently understood (Engelke et al., 2003; Havey and Kim, 2021). As a result, part of the genetic variation may be overlooked when collections are analyzed using only nuclear markers. Another limitation of current research is the limited integration of genotype and phenotype. Many studies focus either on molecular structure or on agro-morphological variation. Fewer studies directly test whether genetic groups correspond to phenotypic differences and breeding-oriented performance (Lyngkhoi et al., 2021; Chalbi et al., 2023).

This limitation is especially relevant for gene bank collections. The value of characterization depends not only on detecting diversity but also on translating it into useful information for conservation and breeding (Ochar and Kim, 2023). Approaches that combine molecular and phenotypic data, together with multi-trait evaluation frameworks, can support the identification of promising breeding material (Sinkovič et al., 2025). Work on onion landraces also highlights the role of molecular tools in preserving local identity and supports the need for combined genomic and phenotypic analyses (Delvento et al., 2022; Lyngkhoi et al., 2021). The Slovenian Plant Gene Bank (SRGB) maintains onion accessions that represent important national genetic resources (Sinkovič et al., 2023). These accessions originate from different agro-ecological regions and are likely to capture adaptation to diverse growing conditions, making them relevant for breeding programs focused on regional performance (Sinkovič et al., 2023). However, their cytoplasmic diversity, nuclear genetic structure, and relationship with bulb phenotype have not yet been analyzed together within a single framework.

Open-pollinated accessions often contain substantial variation both within and among populations (Lyngkhoi et al., 2021; Chalbi et al., 2023). Their genetic identity and breeding relevance cannot always be reliably inferred from passport data or morphology alone (Delvento et al., 2022). An integrated analysis combining cytoplasmic markers, nuclear SSR and ILP loci, and multivariate phenotypic data is therefore needed to address this gap. This approach helps determine how genetic diversity is structured within the collection and whether genetic clusters are useful for breeding-oriented selection. We hypothesize that cytoplasmic and nuclear variation represent partly independent components of diversity in onion, and that genetic structure alone provides limited predictive value for phenotypic performance in heterogeneous, open-pollinated germplasm. The aim of this study was to test whether genetic structure corresponds to phenotypic differentiation and to assess the extent to which genetic ancestry explains variation in key agronomic traits in the SRGB onion collection. Specifically, we (i) characterized cytoplasmic polymorphism and nuclear genetic diversity; (ii) described phenotypic variability and assessed its relationship with genetic structure; and (iii) integrated molecular and phenotypic data to evaluate the relevance of genetic structure for breeding-oriented selection. These analyses provide information for conserving and using onion genetic resources from the Slovenian national collection.

## 2 Materials and methods

### 2.1 Plant material and study design

The study was conducted over two consecutive growing seasons (2021 and 2022) at the Infrastructure Center Jablje, Agricultural Institute of Slovenia (AIS), Ljubljana, Slovenia. Onion accessions maintained in the Slovenian Plant Gene Bank (SRGB) were used in both years and grown at the same site under comparable cultivation conditions. The two study phases served different analytical purposes. In 2021, plants were used for molecular sampling and genotyping. In 2022, the collection was characterized phenotypically using the selected descriptor set.

### 2.2 Field establishment and cultivation conditions

The analyzed material consisted of 60 accessions of *A. cepa* L. var. *cepa*. Three reference cultivars were included in the molecular phase of the study: Ptujska rdeča, Holandska rumena, and the hybrid Talon. Details of the 60 SRGB onion accessions, including accession codes, collection origin, collection site, latitude and longitude, accession status, and collection source, are provided in **Supplementary Table 1**. The three reference cultivars included in the molecular analysis are also listed in **Supplementary Table 1**, together with their cultivar type and seed supplier/source. In both years, the collection was established as a germplasm characterization trial rather than a formally replicated field experiment due to limited seed availability. Seedlings were raised in 160-cell trays in a heated greenhouse. Sowing was conducted at the end of February. Transplanting occurred in mid-April, and harvest was carried out at the end of July. Plants were grown in four-row strips with 30 × 10 cm spacing under drip irrigation. Fertilization included 120 kg ha⁻¹ K_2_O before planting and 28 kg ha⁻¹ N before transplanting. An additional 54 kg ha⁻¹ N was applied during plant growth. Daily meteorological data for 2021 and 2022 were obtained from the weather station nearest the experimental site. These data were summarized by month to describe environmental conditions during the study. Monthly means were calculated for solar radiation, relative humidity, and minimum and mean air temperature, while precipitation was expressed as the monthly total (**Supplementary Figure 1**).

### 2.3 Molecular sampling and DNA extraction/genotyping

#### 2.3.1 Molecular sampling

For DNA-based molecular analyses, young leaf samples were collected in mid-May 2021. Four plants per accession were randomly selected from the established field population (approximately 20 plants per accession), resulting in 240 sampled individuals. From each selected plant, approximately 60–100 mg of young, healthy leaf tissue was collected using sterile forceps. Samples were placed individually into labeled 1.5 mL microcentrifuge tubes and stored at −80 °C until DNA extraction. One representative plant from each of the three reference cultivars was also included, bringing the total molecular dataset to 243 genotypes.

#### 2.3.2 DNA extraction and marker genotyping

Total genomic DNA was extracted from frozen leaf tissue using the DNeasy Plant Pro Kit (Qiagen), following the protocol of Pipan et al. (2018) with minor modifications for onion tissue. DNA quality was assessed by 1% agarose gel electrophoresis, and DNA concentration was measured fluorometrically using a Qubit instrument (Thermo Fisher Scientific). DNA was diluted to 20 ng μL⁻¹ before amplification. Nuclear genetic variation was characterized using 21 previously published onion-specific markers, including 15 simple sequence repeat (SSR) markers and six intron length polymorphism (ILP) markers. These markers were selected for reliable amplification and clear polymorphism across diverse onion germplasm. PCR amplification was performed using AccuStart II PCR ToughMix with locus-specific primers. Where applicable, an M13-tailed primer system with fluorescently labeled universal primers was used for fragment detection. Amplification conditions were based on established onion genotyping protocols, with the PCR protocol used for each marker provided in **Supplementary Table 2** (Fischer and Bachmann, 2000; McCallum et al., 2008; Jayaswall et al., 2019). Cytoplasmic variation was assessed using five PCR-based cytoplasmic markers targeting mitochondrial polymorphisms associated with onion cytoplasm types: orfA501, 5′cob_S, 5′cob_N, OPT, and PSAO (Engelke et al., 2003; Bang et al., 2011). Primer sequences, marker type, fluorescent label, PCR protocol or annealing temperature, and literature reference for each marker are provided in **Supplementary Table 2**. Amplification conditions were based on the published protocols, and the PCR program used for each marker is provided in **Supplementary Table 2**. Cytoplasmic marker profiles were scored as binary presence/absence data. PCR products were initially verified on agarose gels. For SSR and ILP loci, allele sizes were determined by fragment analysis. Fluorescently labeled products were pooled where appropriate, mixed with formamide and a ROX 500 internal size standard, denatured, and analyzed using an ABI 3500 genetic analyzer (Applied Biosystems/Thermo Fisher Scientific). Electropherograms were scored using GeneMapper version 6.0. Nuclear markers were treated as codominant loci, while cytoplasmic markers were treated as dominant binary variables.

### 2.4 Phenotypic evaluation

#### 2.4.1 Phenotypic *material and evaluated subset*

Phenotypic evaluation was conducted in 2022 on plants grown at the same experimental site under the cultivation conditions described above. The trial included the 60 SRGB accessions used in the molecular analysis. The three reference cultivars were not included in this phase. Each accession was represented by ten plants in the field. Two accessions (ALL-50 and ALL-75) did not germinate and were excluded. This resulted in a final phenotypic dataset of 58 accessions. Bulbs were harvested at technological maturity and dried for several weeks in a dark, well-ventilated environment. Only cleaned and fully developed bulbs were used for evaluation. Five bulbs per accession were selected for phenotypic assessment. Because the phenotypic evaluation was conducted as a germplasm characterization trial, no replicated field plots were established. Therefore, environmental variance and plot-level experimental error could not be estimated separately. The five bulbs evaluated per accession were treated as within-accession subsamples. They were used to describe accession-level phenotypic variation. All accessions were grown in the same field, in the same season, and under the same management conditions to reduce field-related variation. Genotype-phenotype analyses were performed using the 58 accessions represented in both datasets.

#### 2.4.2 Phenotypic descriptors and measurements

A total of 47 phenotypic descriptors were recorded, including 39 qualitative and 8 quantitative traits, following the onion descriptor guidelines of UPOV (2008) and CPVO (2009). The quantitative traits were bulb weight (BW), bulb height (BH), bulb diameter (BD), bulb neck width (BNW), height-to-diameter ratio (HDR), total soluble solids (TSS), number of fleshy scale leaves (NSL), and dry matter content (DMC). A complete list of descriptors, scoring classes, and measurement units is provided in **Supplementary Tables 3A–C**. Qualitative descriptors were recorded in two forms. Traits with ordered categorical classes, such as foliage attitude, foliage waxiness, bulb size, and color intensity, were scored directly using the discrete ordinal scales defined in the UPOV and CPVO protocols. Several multi-state morphological descriptors were also recorded, including position of maximum diameter, bulb shape, shape of the stem end, shape of the root end, base color of dry skin, and epidermis color of fleshy scales. Quantitative traits were measured using standard laboratory instruments. Bulb height (BH), bulb diameter (BD), and bulb neck width (BNW) were measured with a digital caliper (Mitutoyo 500-181-30; accuracy 0.1 mm). Bulb weight (BW) was measured using a precision balance (PB1502, Mettler Toledo; accuracy 0.01 g). Total soluble solids (TSS) were determined from freshly extracted onion juice using a portable digital refractometer (WZB-F35, Sumer Instrument, Thermo Shaker) and expressed as °Brix. Small pieces of fresh onion tissue were pressed, and the extracted juice was analyzed in three technical replicates. Number of fleshy scale leaves (NSL) was determined by manual counting. Dry matter content (DMC) was calculated as the percentage of dry mass relative to fresh mass. Fresh bulb tissue was first weighed, then freeze-dried, and weighed again to obtain dry mass.

#### 2.4.3 Phenotypic *data preparation*

Quantitative trait values were averaged across the five bulbs evaluated for each accession. These values were then used in the statistical analyses. Before analysis, the quantitative variables were standardized, allowing traits measured on different scales to be compared more directly. Qualitative descriptors were handled according to their scoring type. Traits recorded with ordered descriptor classes were retained as ordinal variables, while multi-state morphological descriptors were converted into binary presence/absence variables for statistical analyses. Each possible category was treated as a separate variable (1 = present, 0 = absent). Quantitative traits were used for univariate analyses and genotype-phenotype association tests. Mixed-data multivariate analyses included both quantitative and qualitative descriptors. The Selection Index was calculated from standardized quantitative traits along with selected ordinal descriptors.

### 2.5 Data structure and analytical units

The study included analyses at two biological levels: individual genotypes and accessions. Overall nuclear genetic diversity and genetic relationships were examined at the individual level. Population structure and genotype-phenotype relationships were analyzed at the accession level. For accession-based analyses, molecular data from the four sampled individuals were combined to represent each accession. These accession-level profiles included the ancestry coefficients used in integrative analyses.

### 2.6 Genetic data analysis

Cytoplasmic and nuclear marker data were analyzed separately due to their distinct modes of inheritance. Cytoplasmic markers were treated as binary presence/absence variables. For each locus, band frequency (p) was calculated as the proportion of individuals showing band presence. The alternative allele frequency was defined as q = 1 – p, assuming a dominant marker system. Diversity parameters, including the effective number of alleles (Ne), Shannon’s information index (I), and Nei’s gene diversity (h), were calculated using GenAlEx version 6.4.1 (Peakall and Smouse, 2006, Peakall and Smouse, 2012). Monomorphic loci were excluded from mean estimates. As these markers represent diagnostic cytoplasmic variants, diversity values were interpreted as measures of cytoplasmic polymorphism rather than classical population genetic diversity. Genetic relationships based on cytoplasmic markers were assessed using UPGMA clustering. Pairwise dissimilarities were calculated using Jaccard distance from the binary matrix (vegdist function, *vegan* package in R). Clustering was performed using the average-linkage method (*hclust*), and dendrograms were visualized using the *ape* package. Branch support was evaluated by bootstrap resampling with 500 replicates. Nuclear genetic diversity was assessed at the individual genotype level. For each SSR and ILP locus, standard diversity parameters were calculated, including number of alleles (Na), observed heterozygosity (Ho), expected heterozygosity (He), probability of identity (PI), and polymorphic information content (PIC), using GenAlEx version 6.4.1 (Peakall and Smouse, 2006, Peakall and Smouse, 2012).

Genetic differentiation among and within accessions was evaluated using Analysis of Molecular Variance (AMOVA) implemented in the *pegas* package in R (Paradis, 2010). The analysis was based on pairwise genetic distances derived from SSR and ILP loci, calculated as squared differences in allele sizes and averaged across loci, consistent with a stepwise mutation model. Statistical significance was assessed using 10,000 permutations. Population structure within the SRGB collection was inferred using the Bayesian clustering algorithm implemented in STRUCTURE version 2.3.2.1 (Pritchard et al., 2000; Falush et al., 2003). Analyses were performed under the admixture model with correlated allele frequencies. Values of K from 2 to 9 were tested. For each K, four independent runs were conducted with a burn-in of 10,000 iterations followed by 1,000,000 Markov chain Monte Carlo (MCMC) iterations. The most likely number of clusters was determined using the ΔK method of Evanno et al. (2005) implemented in Structure Harvester (Earl and vonHoldt, 2012). STRUCTURE outputs were summarized at the accession level, and ancestry coefficients were used in subsequent analyses. Bar plots of ancestry coefficients were generated in R. Genetic relationships among genotypes and accessions were further explored using Principal Coordinates Analysis (PCoA) and UPGMA clustering based on Nei’s genetic distance. PCoA was performed in GenAlEx. The resulting coordinates were exported and visualized in R using *ggplot2*. UPGMA clustering was conducted using the average-linkage method (*hclust*), and dendrograms were visualized using the *ape* package.

### 2.7 Phenotypic and integrative analyses

Phenotypic differentiation among STRUCTURE-defined nuclear clusters was evaluated using PERMANOVA implemented in the *vegan* package in R (Anderson, 2001). Euclidean distances were calculated from standardized quantitative traits. Statistical significance was assessed using 999 permutations. To test whether differences among clusters were influenced by unequal within-group variability, multivariate homogeneity of dispersion was evaluated using the betadisper function in the *vegan* package in R, followed by analysis of variance. Differences among clusters for individual traits were further assessed using one-way analysis of variance (ANOVA), and effect sizes were expressed as η². False discovery rate (FDR) correction was applied using the Benjamini–Hochberg method (Benjamini and Hochberg, 1995). To summarize the overall phenotypic structure of the collection, Factor Analysis of Mixed Data (FAMD) was performed using both quantitative and qualitative descriptors (*FactoMineR* package; Husson et al., 2017; Lê et al., 2008). This approach allowed joint analysis of continuous and categorical variables and was used to identify the main axes of phenotypic variation and their relationship with nuclear genetic clustering. Associations between genetic ancestry and quantitative traits were evaluated using multiple linear regression models based on accession-level ancestry coefficients derived from STRUCTURE analysis. Because ancestry proportions are not independent and sum to one, only two components were included as predictors, with the third treated as the reference. Model outputs and FDR-adjusted results are provided in **Supplementary Table 5**. A composite Selection Index (SI) was calculated from standardized quantitative traits and selected ordinal descriptors, following classical selection index theory (Hazel, 1943; Céron-Rojas and Crossa, 2018). The index was defined as a linear combination of weighted variable values:


$$SI_{i}=\;\sum_{j=1}^{p}b_{j}x_{ij}$$

where x_ij_ represents the standardized value of variable *j* for accession *i*, and b_j_ represents the weight assigned to that variable according to its breeding relevance. The index was based primarily on weighted quantitative traits, while selected ordinal descriptors were included with smaller weights. Only selected ordinal descriptors were included in the index. These were chosen because they were scored on ordered scales, were biologically interpretable, and were not directly redundant with the measured quantitative bulb traits. Differences in SI among STRUCTURE-defined clusters were evaluated using one-way ANOVA followed by Tukey’s honestly significant difference (HSD) test (Abdi and Williams, 2010). The relationship between SI and genetic ancestry was further assessed using linear regression models (SI ~ Q1 + Q2). Detailed results are provided in **Supplementary Table 6** and **Supplementary Table 7**. Unless otherwise stated, all statistical analyses were performed in R (version 4.3.1).

## 3 Results

A total of 243 onion genotypes were analyzed, including 240 individuals from 60 accessions and three reference cultivars. Four individuals were sampled from each accession. Because the dataset included both individual genotypes and accessions, analyses were performed at two levels. Marker diversity statistics were calculated at the individual genotype level AMOVA was also performed at the individual level, with accession identity used as the grouping factor. STRUCTURE, nuclear PCoA, and nuclear UPGMA were performed at the accession level using accession-averaged marker data. Cytoplasmic UPGMA was based on accession-level cytoplasmic profiles for the 60 SRGB accessions. Phenotypic analyses and Selection Index calculations were performed at the accession level for the 58 accessions with phenotypic data.

### 3.1 Cytoplasmic genetic diversity

Cytoplasmic markers revealed only limited cytoplasmic polymorphism in the dataset (marker details are provided in **Supplementary Table 2**). In several landrace accessions, more than one cytoplasmic profile was observed. The five marker systems produced eight scorable cytoplasm-associated bands/loci used for binary analysis. Organellar variation was therefore assessed using eight cytoplasm-associated loci (**Table 1**). These marker systems commonly used to characterize CMS-related cytoplasmic variation. Among the loci analyzed, 5′cob_N was monomorphic across all genotypes. It was therefore excluded from diversity calculations. For the remaining loci, band-presence frequencies ranged from 0.128 (orfA501b) to 0.938 (5′cob_S). The average band-presence frequency was 0.584. Overall diversity across polymorphic loci was low to moderate (mean Ne = 1.581, I = 0.480, h = 0.326). The 5′cob_S band was detected at high frequency. However, these markers do not allow definitive assignment to formal cytoplasmic types. Cytoplasmic marker patterns were found in accessions from different nuclear clusters. No clear cluster-specific cytoplasmic pattern was observed with the marker system used here. Cytoplasmic variation therefore showed no consistent relationship with nuclear genetic structure.

**Table 1:** Binary diversity statistics for PCR-specific cytoplasm-associated markers in 243 onion individual genotypes (240 individuals from 60 accessions and three reference cultivars).

Marker (locus)	Band frequency	p (dominant allele)	q (recessive allele)	Effective number of alleles (Ne)	Shannon’s index (I)	Nei’s gene diversity (h)
orfA501a	0.284	0.284	0.716	1.685	0.597	0.407
orfA501b	0.128	0.128	0.872	1.286	0.382	0.223
5′cob_S	0.938	0.938	0.062	1.131	0.232	0.116
5′cob_N	Monomorphic	–	–	–	–	–
OPTa	0.749	0.749	0.251	1.603	0.563	0.376
OPTb	0.576	0.576	0.424	1.955	0.682	0.488
PSAOa	0.473	0.473	0.527	1.994	0.692	0.499
PSAOb	0.527	0.527	0.473	1.994	0.692	0.499
Mean	0.584	0.584	0.416	1.581	0.480	0.326

Band frequency represents the proportion of individuals showing band presence (1). For these binary loci, p corresponds to band-presence frequency and q = 1 − p. The monomorphic locus (5′cob_N) was not included in the calculation of mean summary statistics.

### 3.2 UPGMA hierarchical grouping of cytoplasmic markers

**Figure 1:**
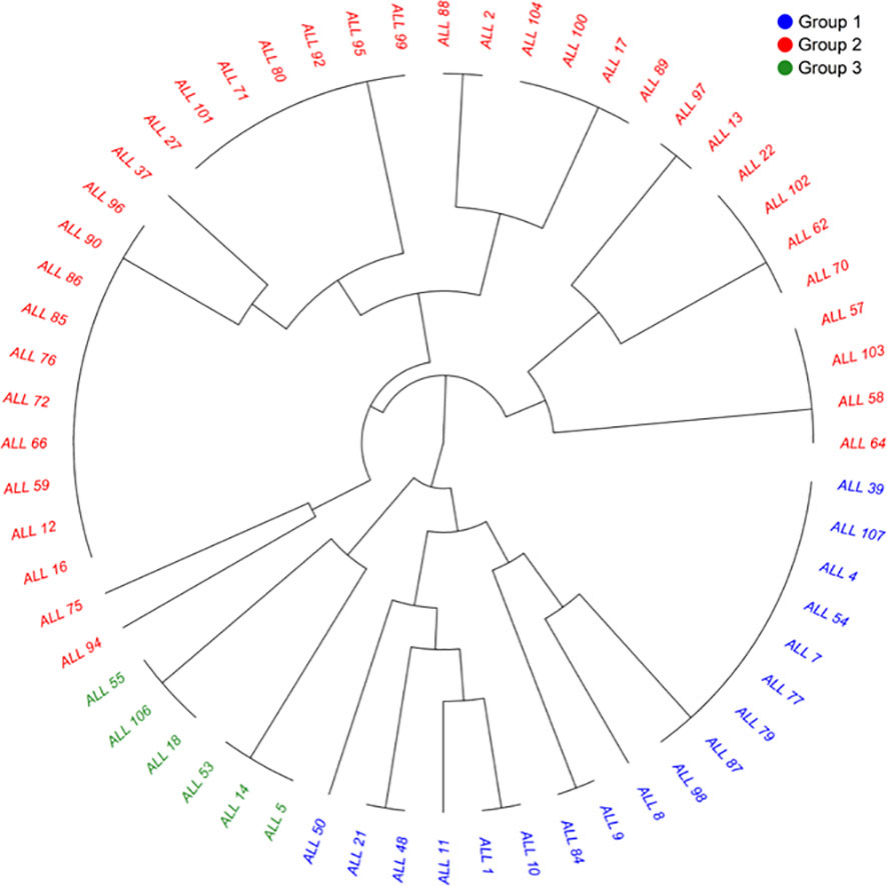
UPGMA dendrogram of cytoplasmic marker profiles in the SRGB onion collection. Grouping was based on eight cytoplasm-associated markers scored as binary presence/absence data. Accession-level cytoplasmic profiles derived from 60 SRGB accessions were used to construct the dendrogram.

The UPGMA dendrogram based on cytoplasmic marker data grouped the accessions into three groups (**Figure 1**). Group 2 (red) contained the most accessions. Group 1 (blue) included fewer accessions than Group 2. Group 3 (green) was the smallest group and contained only a few accessions. These groups reflect different combinations of cytoplasm-associated markers detected in the collection.

### 3.3 Nuclear genetic diversity revealed by SSR and ILP markers

By comparison, the nuclear SSR and ILP loci were highly polymorphic. A total of 312 alleles were detected across the 21 loci analyzed (**Supplementary Table 2**). This corresponded to an average of 14.8 alleles per locus (**Table 2**). The number of alleles ranged from 6 at locus AMS26 to 29 at locus AcILP93. Observed heterozygosity ranged from 0.179 to 0.996 with a mean of 0.821. Expected heterozygosity ranged from 0.630 to 0.898 with a mean of 0.799. Overall, observed heterozygosity was slightly higher than expected heterozygosity. Marker informativeness was high. PIC values ranged from 0.559 to 0.888 with a mean of 0.771. Probability of identity values were consistently low with a mean PI of 0.048.

**Table 2:** Genetic diversity parameters of 21 SSR and ILP loci analyzed in 243 onion individual genotypes (240 individuals representing 60 accessions and three reference cultivars).

Locus	Na	Allele size range (bp)	Ho	He	PI	PIC
AMS08	14	230–274	0.909	0.694	0.051	0.645
AMS23	20	172–396	0.996	0.870	0.051	0.854
AMS25	18	140–494	0.992	0.849	0.051	0.831
AMS12	16	134–462	0.991	0.813	0.045	0.789
AMS17	13	187–365	0.958	0.734	0.045	0.691
AMS21	16	230–274	0.944	0.747	0.045	0.823
AMS26	6	208–220	0.975	0.687	0.051	0.701
AcILP48	8	168–320	0.465	0.764	0.051	0.632
AcILP58	10	154–338	0.823	0.844	0.051	0.732
AcILP112	19	172–396	0.889	0.886	0.051	0.874
AcILP47	16	140–494	0.741	0.846	0.048	0.831
AcILP93	29	134–462	0.770	0.898	0.051	0.888
AcILP103	18	213–495	0.906	0.796	0.038	0.775
ACM033	11	194–270	0.978	0.630	0.038	0.559
ACM038	10	218–330	0.899	0.784	0.037	0.748
ACM045	14	250–348	0.779	0.863	0.048	0.847
ACM054	17	165–321	0.955	0.790	0.051	0.773
ACM058	18	240–350	0.543	0.893	0.051	0.881
ACM077	9	147–417	0.945	0.767	0.049	0.732
ACM094	18	121–243	0.601	0.848	0.051	0.830
ACM147	12	251–307	0.179	0.778	0.048	0.745
Mean	14.8	—	0.821	0.799	0.048	0.771

Na, number of alleles per locus; Ho, observed heterozygosity; He, expected heterozygosity; PI, probability of identity; PIC, polymorphic information content.

### 3.4 Analysis of molecular variance among accessions

AMOVA was used to partition nuclear genetic variation among and within accessions. The analysis included 243 genotypes representing 60 SRGB accessions and three reference cultivars. Most nuclear genetic variation occurred within accessions rather than among them. Specifically, 66.35% of the total variation was within accessions. The remaining 33.65% was found among accessions (**Table 3**). Genetic differentiation among accessions was moderate and statistically significant (ΦST = 0.314, p < 0.001; 10,000 permutations). This indicates measurable nuclear differentiation among accessions.

**Table 3:** Analysis of molecular variance (AMOVA) partitioning nuclear genetic variation among and within accessions based on SSR and ILP markers (n = 243 individual genotypes).

Source of variation	df	SS	MS	Estimated variance	Variation (%)
Among accessions	62	907,210,978	14,632,435	2,511,149	33.65
Within accessions	180	891,154,507	4,950,858	4,950,858	66.35
Total	242	1,798,365,484	7,431,262	7,462,007	100

ΦST = 0.314 (p < 0.001; 10,000 permutations).

### 3.5 Population structure inferred from Bayesian clustering

**Figure 2:**
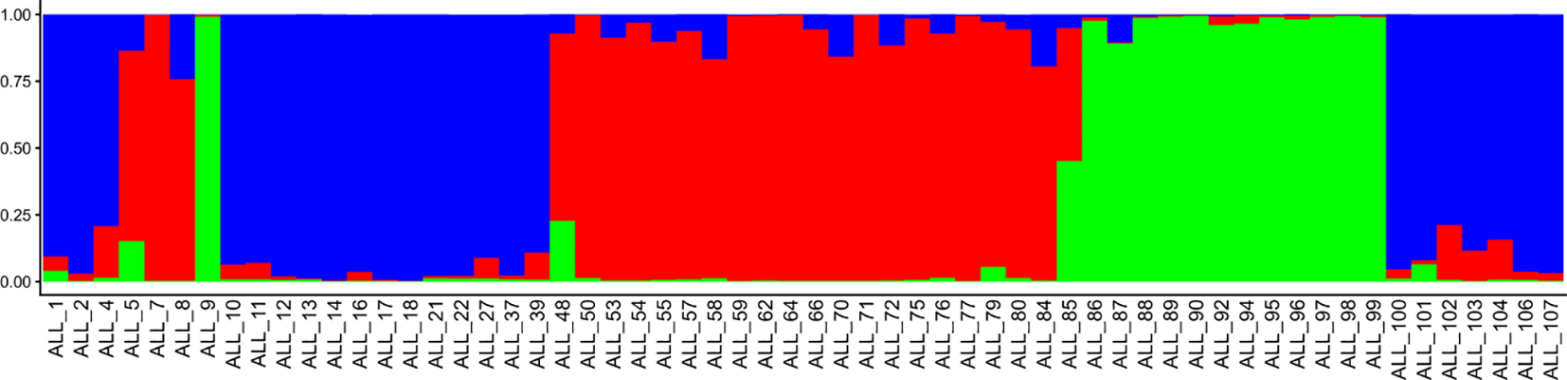
Bayesian population structure of 60 onion accessions inferred from accession-averaged ancestry coefficients (K = 3).

Bayesian STRUCTURE identified three nuclear genetic clusters in the SRGB onion collection (K = 3; **Figure 2**). The analysis was based on accession-averaged ancestry coefficients. The STRUCTURE bar plot showed that most accessions had high membership probabilities for a single cluster. Cluster 1 (blue) included many accessions with high membership values (Q ≈ 0.90–0.99). Cluster 2 (red) included accessions assigned mainly to the second ancestry component. Cluster 3 (green) included fewer accessions, most with very high membership values (Q > 0.95). Only a few accessions showed admixture. These accessions had partial ancestry from two components. Membership coefficients and cluster assignments for all accessions are provided in **Supplementary Table 4**.

### 3.6 Genetic relationships among accessions

#### 3.6.1 Principal coordinates analysis

**Figure 3:**
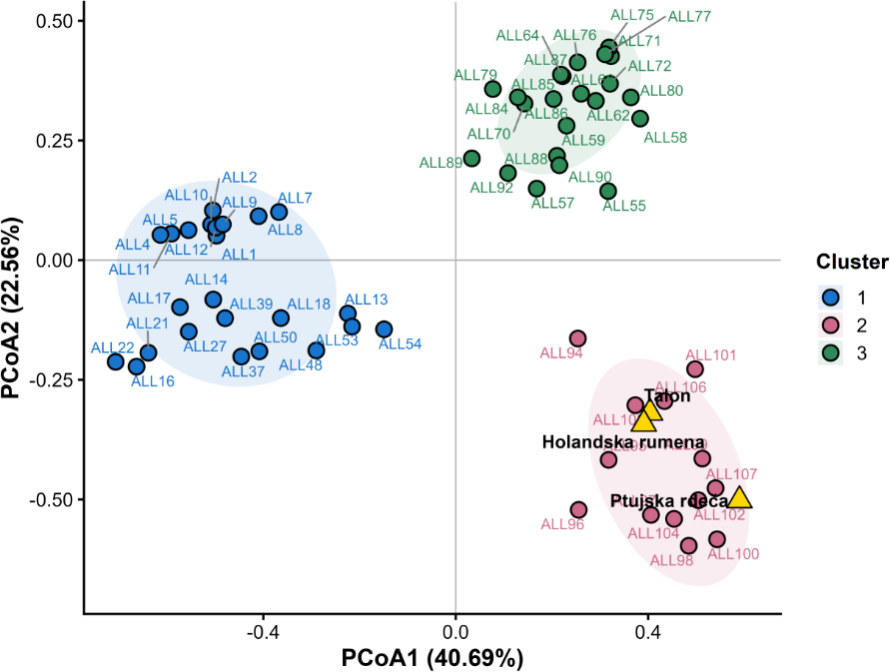
Principal coordinates analysis (PCoA) of 60 SRGB accessions and three reference cultivars based on accession-level SSR and ILP marker data.

Principal Coordinates Analysis (PCoA) was used to examine genetic relationships among accessions (**Figure 3**). The first axis explained 40.69% of the variation. The second axis explained 22.56%. Together, they explained 63.25% of the total variation. The PCoA plot showed three clear clusters. This pattern agreed with the STRUCTURE analysis. Cluster 1 was separated along the first axis, while Clusters 2 and 3 were separated along the second axis. Accessions from the same genetic cluster were generally close to each other. The separation among the three clusters was clear. The reference cultivars Talon, Holandska rumena, and Ptujska rdeča were located within the Cluster 2 region. They grouped close to several SRGB accessions.

#### 3.6.2 UPGMA hierarchical clustering of nuclear markers

**Figure 4:**
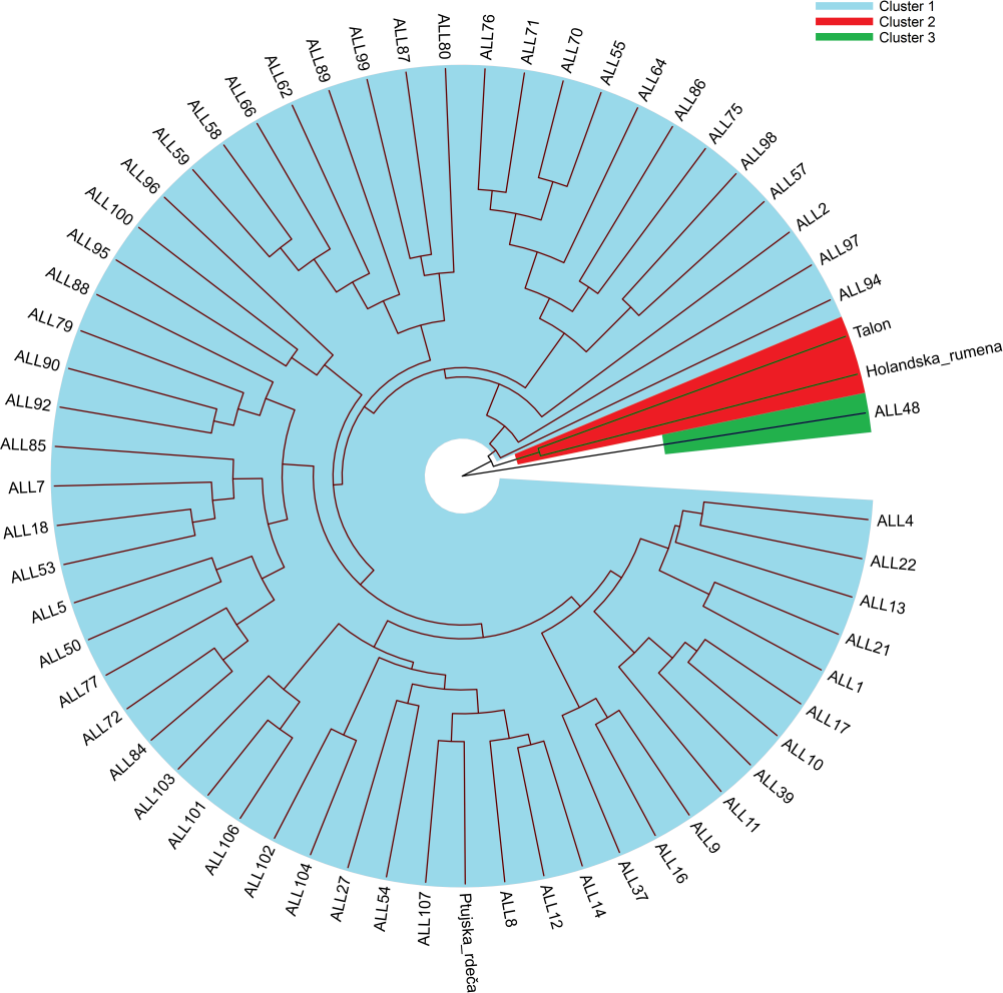
UPGMA dendrogram of nuclear genetic relationships among onion accessions. The dendrogram was constructed from accession-level mean allele sizes of 21 SSR and ILP loci for 60 SRGB accessions and three reference cultivars using Euclidean genetic distances.

UPGMA clustering of nuclear SSR and ILP markers showed genetic relationships among onion accessions (**Figure 4**). The dendrogram included the SRGB accessions along with the three reference cultivars, Talon, Holandska rumena, and Ptujska rdeča. Most SRGB accessions grouped in one large cluster. The reference cultivars Talon and Holandska rumena clustered together as a small separate group. In contrast, accession ALL48 appeared on a separate branch. Overall, the dendrogram showed that most accessions were closely related, while a few accessions or cultivars were more divergent.

### 3.7 Phenotypic differentiation among nuclear genetic clusters

The three nuclear genetic clusters differed significantly in their overall phenotypic profiles (PERMANOVA: R² = 0.274, F = 10.39, p = 0.001; 999 permutations; **Table 4**). Cluster membership explained 27.4% of the total phenotypic variation. Within-cluster variability also differed significantly (F = 12.63, p < 0.001). Thus, the phenotypic differences reflected both cluster separation and differences in variability within clusters. Several quantitative traits differed significantly among the three clusters (**Table 5**). Trait definitions and scoring scales are provided in **Supplementary Tables 3A–C**. Bulb weight showed the strongest difference among clusters (η² = 0.603, FDR-adjusted p < 0.001). This was followed by bulb height (η² = 0.422) and bulb neck width (η² = 0.372). Bulb diameter also showed a clear difference among clusters (η² = 0.280). The height-to-diameter ratio showed a weaker difference (η² = 0.204). Total soluble solids also differed among clusters, but the effect was smaller (η² = 0.157, FDR-adjusted p = 0.012). Dry matter content showed a similar weak but significant difference (η² = 0.115, FDR-adjusted p = 0.040). In contrast, the number of fleshy scale leaves showed no significant differences among clusters (η² = 0.041).

**Table 4:** Multivariate differentiation and dispersion among nuclear clusters.

Test	Source	Df	R²	F	p-value
PERMANOVA	Cluster	2	0.274	10.39	0.001
PERMANOVA	Residual	55	0.726	–	–
Dispersion (betadisper)	Cluster	2	–	12.63	<0.001

PERMANOVA was based on Euclidean distances of standardized quantitative traits (999 permutations). Dispersion was assessed by ANOVA on multivariate homogeneity of variances.

**Table 5:** Trait-wise phenotypic differentiation among nuclear clusters.

Trait	F	η²
Bulb weight	41.74	0.603^**^
Bulb height	20.04	0.422^**^
Neck width	16.29	0.372^**^
Bulb diameter	10.71	0.280^**^
Height/diameter ratio	7.04	0.204^**^
Total soluble solids	5.11	0.157^*^
Dry matter content	3.57	0.115^*^
No. of fleshy scales	1.18	0.041^ns^

η² represents proportion of variance explained by nuclear cluster. ^**^FDR-adjusted p < 0.01; ^*^FDR-adjusted p < 0.05; ^ns^, not significant.

### 3.8 Multivariate phenotypic structure

**Figure 5:**
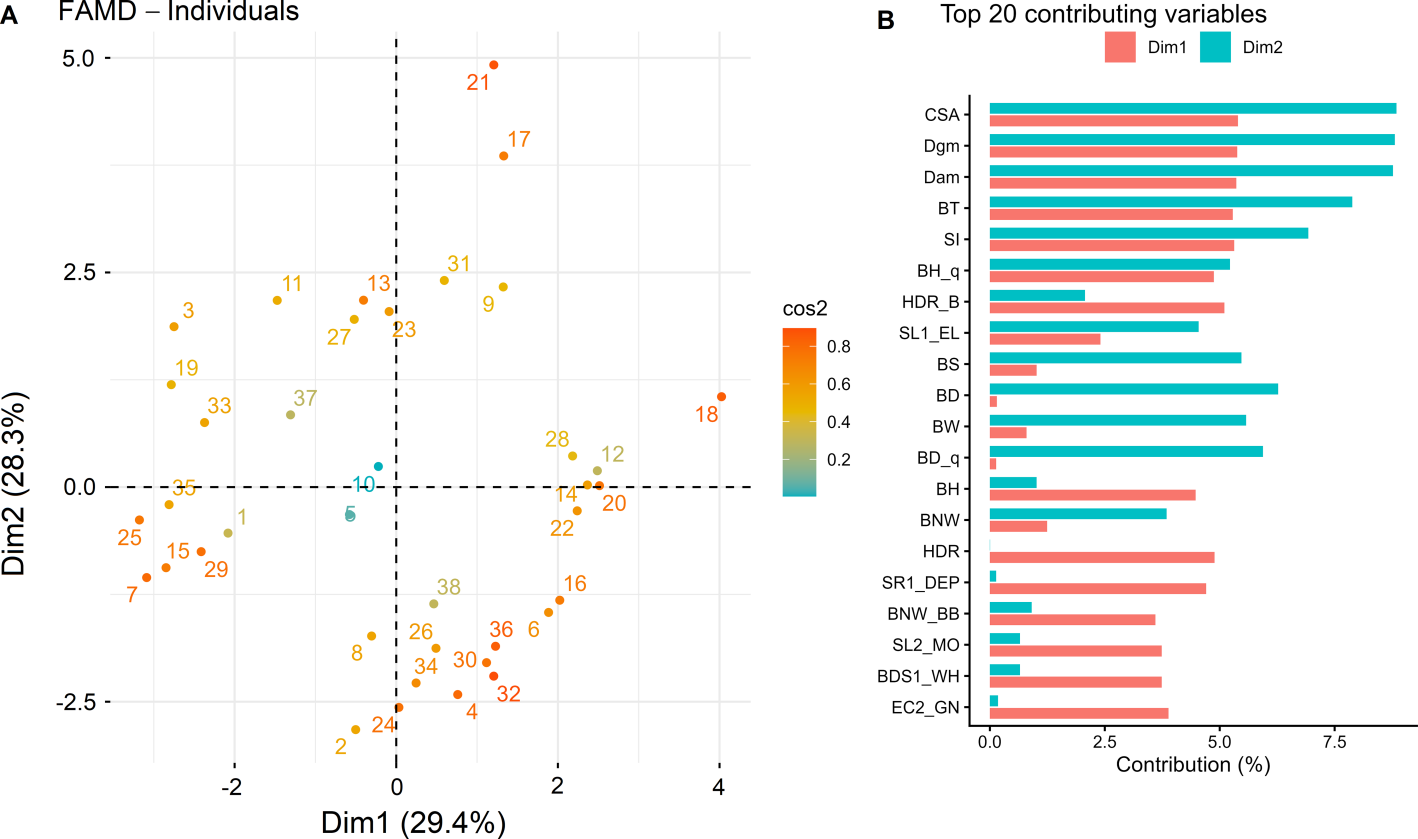
Multivariate phenotypic structure of onion accessions revealed by FAMD. **(A)** Projection of accessions onto the first two FAMD dimensions (Dim1 and Dim2). **(B)** Contribution (%) of the top phenotypic descriptors to Dim1 and Dim2. Trait abbreviations are defined in **Supplementary Tables 3A–C**.

**Figure 6:**
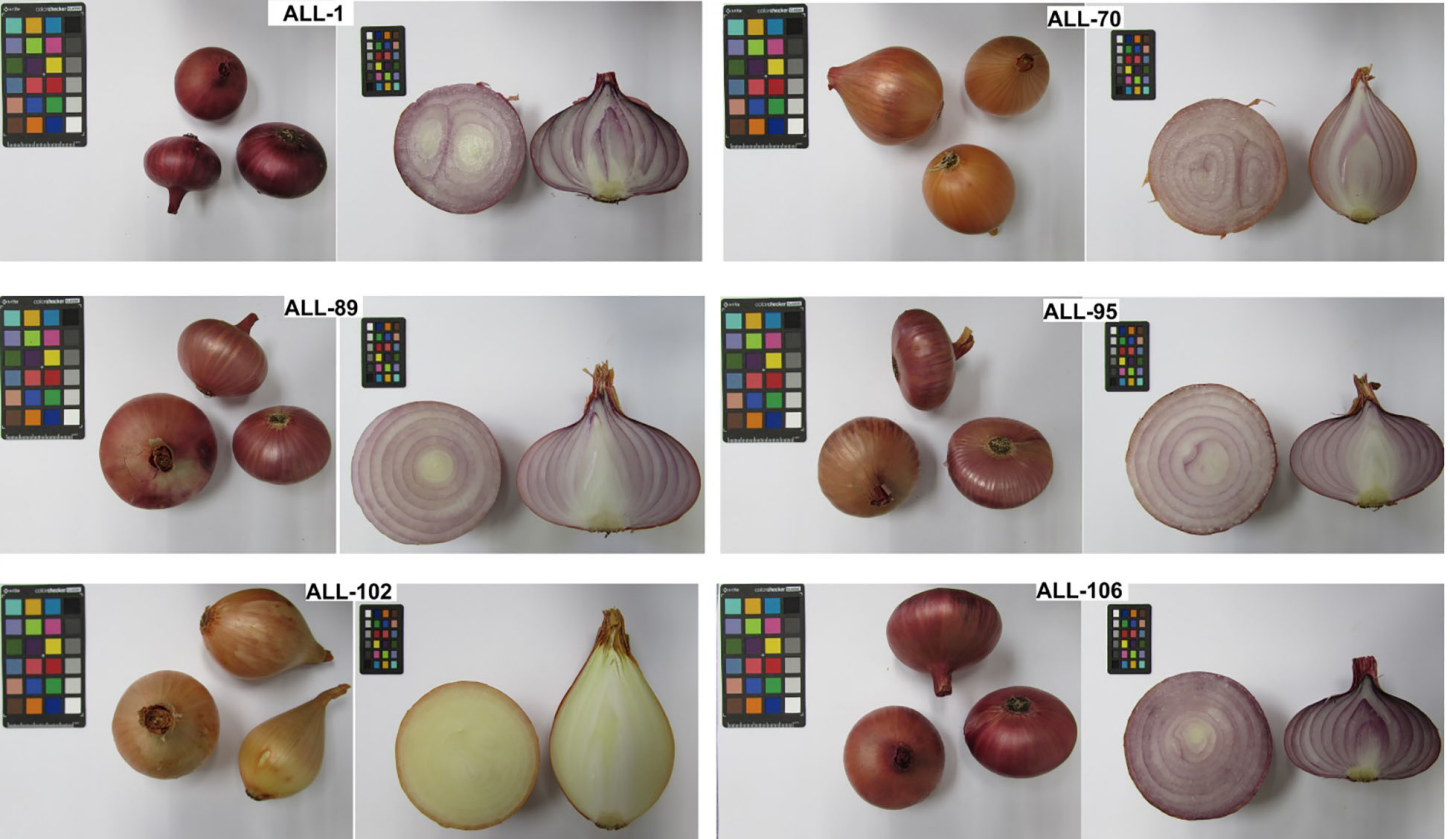
Representative images of selected SRGB onion accessions showing variation in bulb morphology. The images show external bulb appearance and bulb sections for six accessions. Visible differences were observed in bulb shape, dry skin color, and internal structure. The selected accessions represent contrasting morphologies observed in the SRGB onion collection.

FAMD revealed a continuous pattern of phenotypic variation among accessions (**Figures 5A, B**). Representative bulb images illustrate the visible variation in bulb size, shape, dry skin color, and internal structure among selected SRGB onion accessions (**Figure 6**). In the ordination plot (**Figure 5A**), accessions were widely distributed and did not form clear phenotypic groups. The first two dimensions explained 29.4% (Dim1) and 28.3% (Dim2) of the total phenotypic variation. Together they explained 57.7% of the variation. Trait contribution analysis (**Figure 5B**) showed that Dim1 was mainly related to plant architecture and bulb shape. The main contributing traits were bulb height, height-to-diameter ratio, bulb neck width, and several categorical shape traits. Accessions with negative Dim1 values tended to have taller plants and narrower bulbs. Accessions with positive Dim1 values were associated with more compact bulb architecture. Dim2 was more strongly related to bulb size and shape measurements. The main contributing traits were bulb diameter, bulb weight, and related derived measurements. In contrast, total soluble solids and dry matter content contributed only weakly to the first two dimensions.

### 3.9 Association between genetic ancestry and phenotypic traits

**Figure 7:**
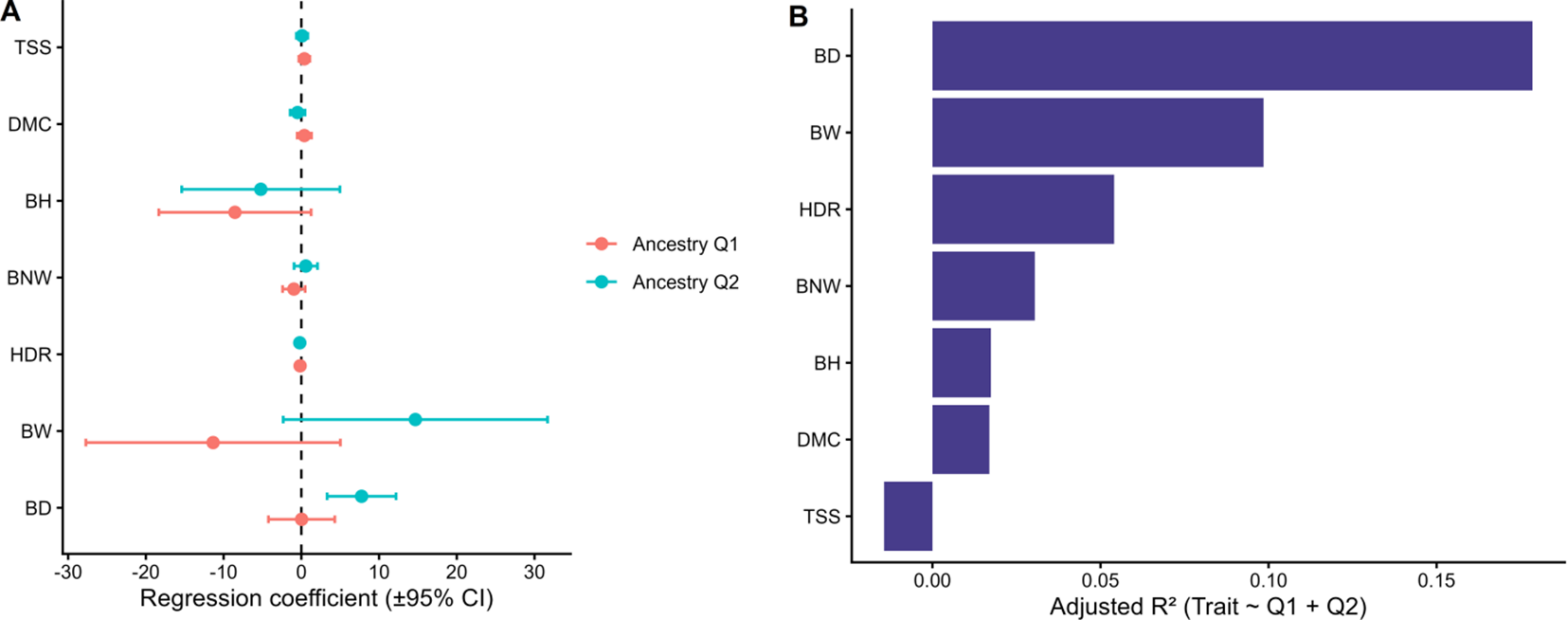
Effects of genetic ancestry components on phenotypic traits. **(A)** Regression coefficients (± 95% confidence intervals) of ancestry components Q1 (red) and Q2 (blue) from multiple linear regression models (Trait ~ Q1 + Q2). The dashed vertical line indicates zero effect. **(B)** Adjusted R² values showing the proportion of phenotypic variance explained jointly by ancestry components for each trait.

**Figure 8:**
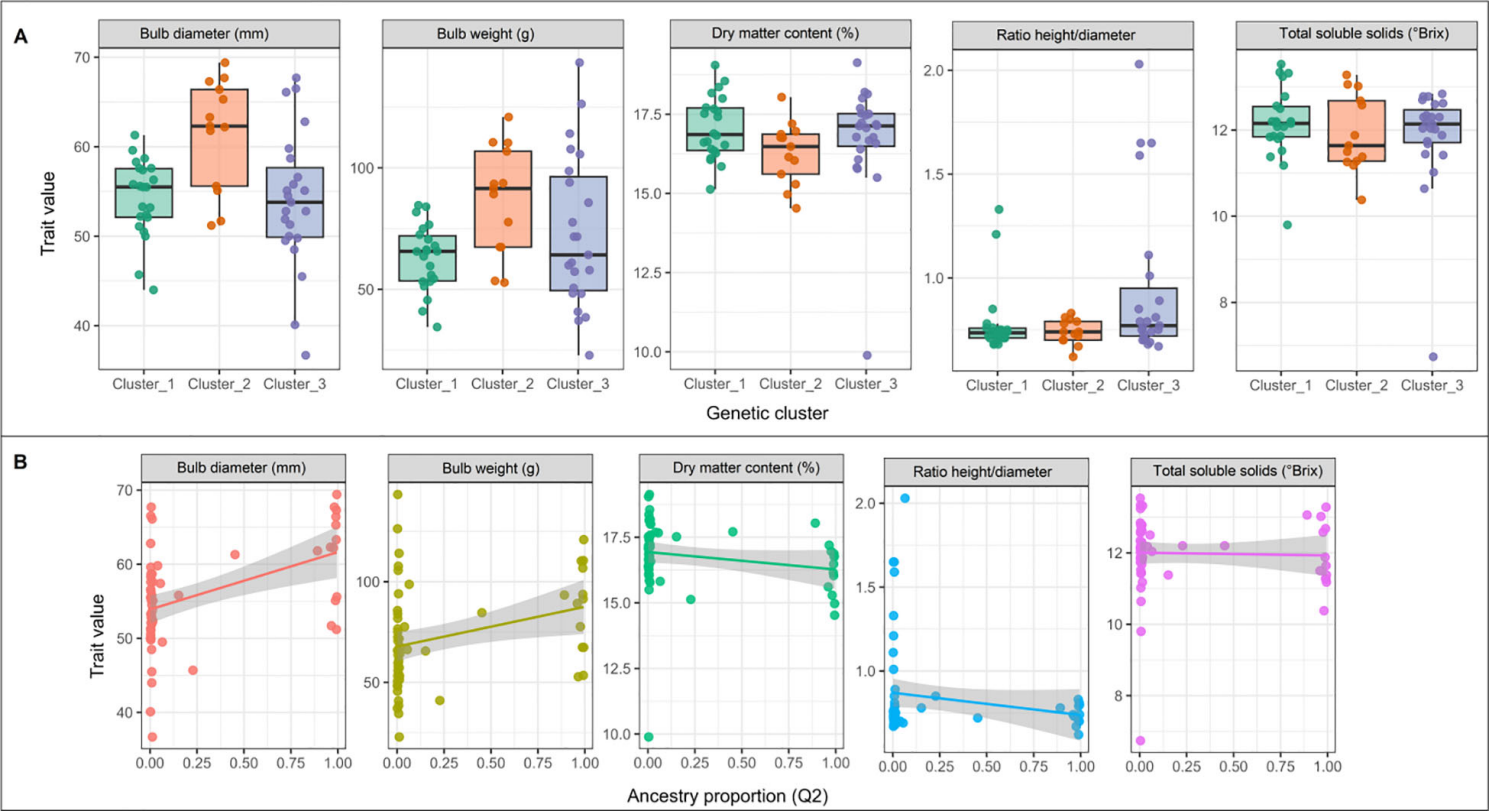
**(A)** Distribution of Bulb diameter (mm), Bulb weight (g), Dry matter content (%), Ratio height/diameter and Total soluble solids (°Brix) across STRUCTURE-derived genetic clusters (K = 3). Boxplots show medians, interquartile ranges, and individual accessions. **(B)** Relationships between Q2 ancestry proportion and the same traits. Solid lines represent fitted linear regressions with 95% confidence intervals.

Overall, regression models that included ancestry coefficients explained only a small proportion of phenotypic variation. Among the analyzed traits, bulb diameter showed the strongest association with ancestry component Q2 (**Figure 7****;** **Supplementary Table 5**). This trait showed the highest explained variance (R² = 0.207; adjusted R² = 0.178). Bulb weight showed a weaker association (R² = 0.130; adjusted R² = 0.098). Other traits showed very low explained variance. In the regression model, Q2 was positively associated with bulb diameter (β = 7.75 ± 2.26; p = 0.0012). This association remained significant after FDR correction (FDR p = 0.008). No significant ancestry effects were detected for BNW, BH, DMC, or TSS. The negative association between Q2 and the height-to-diameter ratio was weak. It was also not significant after correction. Phenotypic patterns across STRUCTURE-derived clusters supported the regression results (**Figures 8A, B**). In the boxplots, cluster 2 generally showed higher median values for bulb diameter and bulb weight than the other clusters (**Figure 8A**). In contrast, dry matter content, height-to-diameter ratio, and total soluble solids largely overlapped among clusters.

### 3.10 Identification of superior accessions using the Selection Index

**Figure 9:**
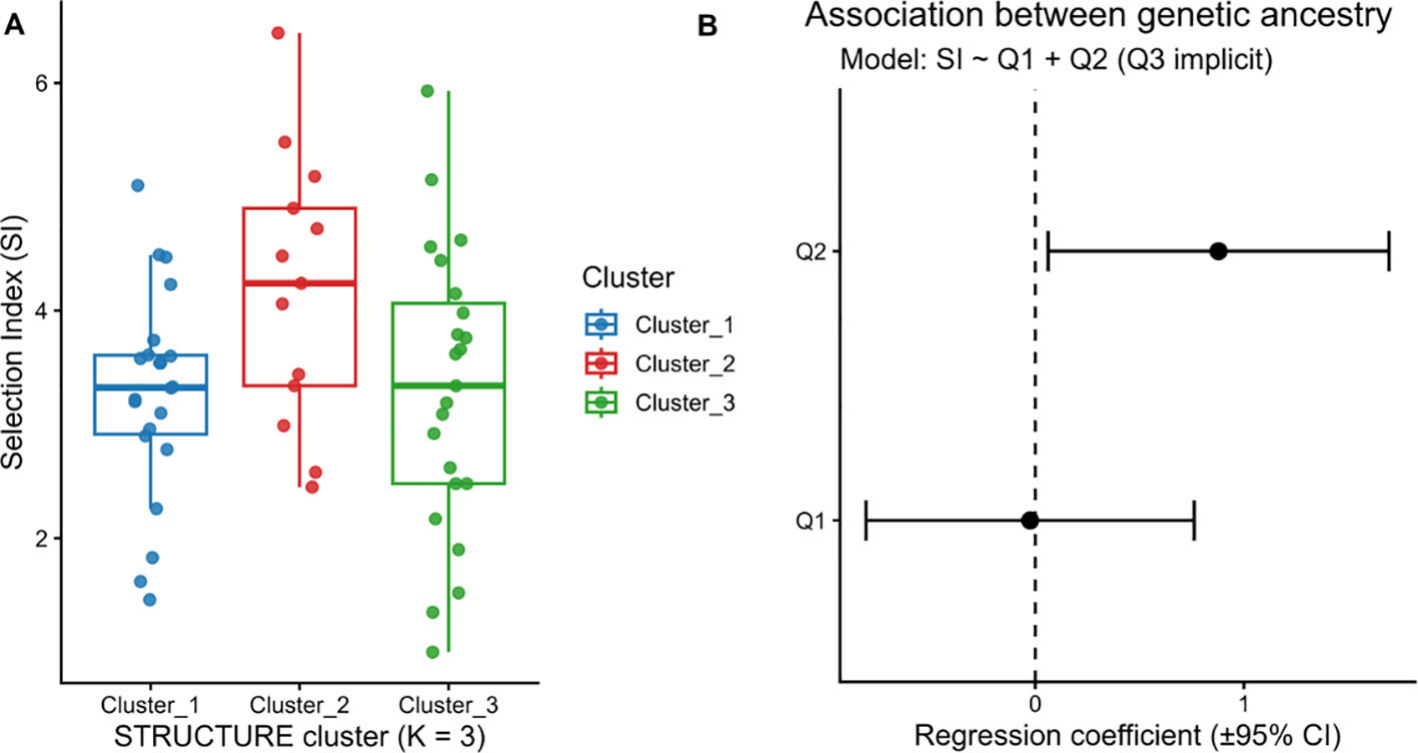
Relationship between Selection Index and nuclear genetic structure (K = 3). **(A)** Distribution of Selection Index (SI) across STRUCTURE-defined clusters (n = 58). Differences were tested using one-way ANOVA (F_2_,_55_ = 3.25, p = 0.046). **(B)** Regression coefficients (± 95% CI) from the model SI ~ Q1 + Q2 (Q3 implicit). Q2 showed a significant positive association with SI (p = 0.036), whereas Q1 was not significant. Model statistics: R² = 0.096; adjusted R² = 0.063; p = 0.062.

Selection Index values varied substantially among accessions. High-ranking accessions were found in all three STRUCTURE-defined clusters. A significant overall effect of cluster on SI was detected (ANOVA: F_2_,_55_ = 3.25, p = 0.046; **Supplementary Table 7A**). Cluster 2 showed the highest median SI value (**Figure 9A**). However, Tukey pairwise comparisons showed no significant differences between cluster pairs (**Supplementary Table 7B**). Regression analysis indicated a positive association between SI and ancestry component Q2 (β = 0.88 ± 0.41, p = 0.036). In contrast, Q1 was not significantly associated with SI (**Figure 9B****;** **Supplementary Table 7C**). The overall model explained only a small proportion of SI variation (R² = 0.10; adjusted R² = 0.06). However, the model was not statistically significant overall (F_2_,_55_ = 2.93, p = 0.062; **Supplementary Table 7C**). These results show that SI was not determined by cluster membership alone. Breeding-oriented potential was distributed across the collection, including within each genetic cluster.

## 4 Discussion

This study indicates that the SRGB onion collection is structured into distinct nuclear genetic groups, and that these groups are associated with broad differences in bulb morphology. However, genetic structure explained only part of the phenotypic variation and did not define a single superior group for selection. This supports accession-level evaluation when using SRGB onion germplasm for breeding.

### 4.1 Cytoplasmic diversity and its weak correspondence with nuclear structure

Cytoplasmic marker analysis revealed low but detectable organellar polymorphism in the SRGB onion collection (**Table 1**). This aligns with the generally lower diversity of cytoplasmic genomes, reflecting maternal inheritance and limited recombination. Similar findings have been reported in other onion germplasm collections. In those studies, cytoplasmic variation was substantially lower than the diversity detected by nuclear SSR markers (Engelke et al., 2003; Havey and Kim, 2021; Khrustaleva et al., 2023). The relatively high frequency of the 5′cob_S band indicates that S-associated mitochondrial polymorphisms are common in the analyzed germplasm (**Table 1**). However, the marker set used here detects cytoplasmic polymorphism rather than precisely distinguishing among cytoplasmic types. Such discrimination typically requires multilocus diagnostic systems combining several mitochondrial loci (Engelke et al., 2003). Cytoplasmic polymorphism does not necessarily correspond directly to male sterility. Expression of cytoplasmic male sterility depends on interactions between the mitochondrial cytoplasm and nuclear restorer-of-fertility (Rf) genes. These interactions form the basis of hybrid onion breeding systems (Havey and Kim, 2021). In this study, cytoplasmic marker patterns showed no clear correspondence with nuclear genetic clusters (**Figures 2**, **3**) inferred from SSR and ILP markers. This suggests that organellar and nuclear variation are largely independent, although some associations cannot be excluded. This is consistent with the maternal inheritance of cytoplasmic genomes and pollen-mediated gene flow affecting nuclear diversity in the outcrossing onion reproductive system (Havey and Kim, 2021). From a breeding perspective, the widespread occurrence of S-associated polymorphisms in the SRGB collection is relevant. Cytoplasmic male sterility remains central to hybrid onion production. While cytoplasmic markers revealed only limited polymorphism, analysis of nuclear markers provided a much more detailed view of genetic diversity and differentiation within the SRGB collection.

### 4.2 Nuclear genetic diversity and accession-level differentiation

The present study demonstrates that the SRGB onion collection contains substantial nuclear genetic variation and clear accession-level differentiation. Nuclear SSR and ILP markers revealed high polymorphism and strong discriminatory power (**Table 2**), confirming that the collection represents a diverse germplasm resource. Observed heterozygosity was slightly higher than expected heterozygosity, consistent with the high heterozygosity typically found in outcrossing species such as onion and with the heterogeneous composition of open-pollinated landraces (Lyngkhoi et al., 2021). Comparable levels of genetic diversity have also been reported in other onion germplasm collections analyzed with molecular markers (Chalbi et al., 2023). AMOVA further showed that most molecular variation resides within accessions (**Table 3**), while a substantial proportion is also partitioned among accessions (Excoffier et al., 1992; Lyngkhoi et al., 2021; Chalbi et al., 2023). The distribution of genetic variation observed in the SRGB accessions is typical of open-pollinated landraces. In these populations, outcrossing maintains considerable within-population diversity, while genetic drift, farmer selection, and partially restricted seed exchange contribute to divergence among populations. In practical terms, the SRGB accessions can be regarded as internally variable but genetically differentiated populations. This has direct implications for germplasm management. Regeneration schemes should maintain sufficiently large and balanced numbers of plants to minimize genetic drift while preserving accession identity. Similar recommendations have been emphasized in recent syntheses on onion germplasm conservation and utilization (Ochar and Kim, 2023). Considering nuclear diversity, cytoplasmic profiles, phenotypic structure, ancestry-trait relationships, and Selection Index results together provides a more complete view of diversity in the SRGB onion collection. Together, these results show that the collection contains both within-accession diversity and clear differentiation among accessions.

### 4.3 Genetic structure and complementary insights from UPGMA

STRUCTURE identified three nuclear genetic clusters (**Figure 2**), and the PCoA showed the same overall pattern (**Figure 3**). In the ordination, the three clusters occupied distinct regions, indicating consistent genetic subdivision within the SRGB collection. UPGMA clustering (**Figure 4**) provided a complementary perspective on genetic relationships. Most SRGB accessions were located within a large cluster, reflecting the overall genetic relatedness of much of the material, but the dendrogram also highlighted distinct genetic divergence among certain genotypes. In particular, the reference cultivars Talon and Holandska rumena formed a small separate cluster, and accession ALL48 appeared on an isolated branch, indicating a higher level of genetic differentiation. Overall, the clustering pattern suggests that the collection is composed primarily of related accessions, along with a smaller number of more differentiated genotypes. The agreement between the model-based STRUCTURE analysis (**Figure 2**) and the ordination-based PCoA (**Figure 3**) supports this pattern. This supports K = 3 as a biologically meaningful level of subdivision, although alternative structures cannot be entirely excluded (Pritchard et al., 2000; Evanno et al., 2005). Although the PCoA revealed well-defined clusters, some proximity among accessions from different clusters was still observed, which is expected in an outcrossing crop and is consistent with the substantial within-accession diversity detected by AMOVA (**Table 3**). These results indicate that the accessions are genetically coherent but still heterogeneous populations, consistent with previous onion germplasm studies (Lyngkhoi et al., 2021; Chalbi et al., 2023).

The historical drivers of this subdivision cannot be fully resolved here. The three nuclear clusters most likely reflect historical divergence shaped by breeding history, partial isolation among seed sources, and local production conditions (Delvento et al., 2022; Monteverde et al., 2015; Lyngkhoi et al., 2021; Chalbi et al., 2023). In an outcrossing species such as onion, the persistence of distinct nuclear clusters despite ongoing gene flow is consistent with partially connected seed exchange and breeding systems. Genetic exchange may occur without fully erasing accumulated divergence, particularly when seed exchange is partial or structured (Chalbi et al., 2023; Monteverde et al., 2015). In the SRGB collection, this structure highlights the importance of maintaining effective population size during regeneration and avoiding unintended admixture among genetically differentiated accessions. In the SRGB material, the presence of three nuclear clusters also provides a practical framework for crossing strategies. Inter-cluster crosses may increase genetic divergence and broaden breeding variation, whereas within-cluster selection can exploit the substantial diversity retained within individual populations (Falconer and Mackay, 1996).

### 4.4 Phenotypic differentiation associated with genetic structure

Nuclear genetic structure was associated with significant phenotypic differentiation among accessions (**Table 5**). In the multivariate analysis, cluster membership explained 27.4% of the total phenotypic variance (**Table 4**), although significant differences in multivariate dispersion indicate that this signal reflects both differences in cluster centroids and variation within clusters. The most strongly differentiated traits were bulb weight, bulb height, bulb neck width, and bulb diameter (**Table 5**). These traits also contributed substantially to the main phenotypic axes identified by FAMD (**Figure 5**). They are directly related to bulb architecture, market class, and agronomic value. More specifically, Cluster 2 was associated with larger bulb diameter and higher bulb weight. This pattern was also supported by the positive relationship between Q2 ancestry and bulb diameter. Therefore, the main phenotypic signal linked to nuclear structure was related to bulb architecture rather than composition. This may reflect past selection or seed maintenance for bulb size in part of the collection. In contrast, total soluble solids and dry matter content showed weaker differentiation among clusters. These traits are influenced by both genotype and environment. They can vary with water availability, soil fertility, temperature, harvest maturity, and post-harvest drying conditions (Sekara et al., 2017; Kazimierczak et al., 2021). In this study, they showed weaker differentiation among nuclear genetic clusters than bulb size and morphology traits. Multi-year and multi-location evaluation would help determine their stability across environments. This pattern is consistent with previous work showing that onion compositional and quality traits are strongly influenced by environmental conditions and agronomic management (Sekara et al., 2017; Kazimierczak et al., 2021; Chalbi et al., 2023). FAMD further supported this interpretation by identifying two major phenotypic axes (**Figure 5**). The first was mainly related to plant architecture and bulb proportions, while the second was more strongly associated with bulb size and external appearance. The partial but overlapping separation of STRUCTURE-defined clusters in phenotypic space suggests that nuclear genetic structure reflects broad trends in bulb morphology, but does not define clearly discrete phenotypic classes. To explore this relationship further, ancestry-based regression models were used to examine how strongly population structure predicts variation in individual quantitative traits.

### 4.5 Structure-trait relationships and limits of ancestry-based prediction

Regression models based on ancestry coefficients showed that broad genetic structure explained only a modest proportion of variation in most quantitative traits (**Figure 7**). The strongest relationships were observed for bulb diameter and, to a lesser extent, bulb weight. After correction for multiple testing, only the positive association between Q2 ancestry and bulb diameter remained significant. This indicates that population structure captures broad phenotypic tendencies but does not fully explain the polygenic architecture of quantitative traits. Because ancestry coefficients summarize genome-wide allele-frequency patterns rather than specific causal loci, this result is consistent with the expected polygenic control and environmental sensitivity of bulb-related traits (Falconer and Mackay, 1996). Similar outcomes have been reported in other onion germplasm studies (Chalbi et al., 2023; Brahimi et al., 2022). The enrichment of larger bulbs in Cluster 2, together with the positive relationship between Q2 ancestry and bulb diameter (**Figures 7**, **8**), indicates that bulb size and architecture differ among nuclear genetic clusters. This pattern suggests that bulb architecture represents one of the principal axes of phenotypic differentiation within the SRGB material. Because bulb diameter and bulb weight contribute positively to marketable bulb yield in onion, repeated selection for bulb size may have contributed to divergence among gene pools (Yeshiwas et al., 2024). In contrast, the weaker structuring of dry matter and soluble solids is consistent with their stronger genotype × environment sensitivity. Overall, these results suggest that genetic structure reflects broad phenotypic tendencies. An additional question is whether this limited predictive power of ancestry is also reflected in the distribution of overall breeding value across the genetic structure of the collection.

### 4.6 Distribution of breeding value across structured diversity

The Selection Index (SI) indicated that breeding value is distributed throughout the genetic structure of the collection (**Table 6****;** **Figure 9**). SI values covered a broad range, and the highest-ranking accessions were present in all three STRUCTURE-defined clusters. Although ANOVA detected a weak overall cluster effect, pairwise comparisons were not significant, and ancestry accounted for only a small proportion of SI variation (**Figure 9**). In cross-pollinated crops such as onion, within-population variance for quantitative traits often exceeds between-population variance, reflecting the heterogeneous composition of open-pollinated populations (Falconer and Mackay, 1996; Lyngkhoi et al., 2021; Brahimi et al., 2022). Therefore, structured divergence does not necessarily mean that breeding value is concentrated in a single cluster. The modest positive relationship between Q2 ancestry and SI parallels the corresponding relationship with bulb diameter and may indicate some enrichment for size-related allelic combinations in that cluster. Thus, Cluster 2 may be useful as a source of larger-bulb material, but high-ranking accessions were still present in all clusters. However, the low explanatory power of the regression model shows that multi-trait performance is not primarily determined by broad population structure, which aligns with expectations for complex quantitative traits (Falconer and Mackay, 1996). High-ranking accessions were found in all three STRUCTURE-defined clusters, indicating that no single genetic cluster contained all top-ranked material. This suggests that superior accessions can be selected from across the collection. These findings have important implications for both the conservation and practical use of onion germplasm maintained in regional gene bank collections.

**Table 6:** Top 10 onion accessions ranked according to the Selection Index (SI), including STRUCTURE-derived cluster assignment (K = 3) and ancestry proportions (Q1, Q2, Q3).

Accession	Cluster	Q1	Q2	Q3	Selection index
ALL_89	Cluster_2	0.004	0.992	0.004	6.44
ALL_106	Cluster_3	0.031	0.006	0.964	5.93
ALL_87	Cluster_2	0.005	0.891	0.104	5.48
ALL_95	Cluster_2	0.005	0.989	0.006	5.18
ALL_17	Cluster_3	0.004	0.003	0.993	5.15
ALL_70	Cluster_1	0.838	0.004	0.158	5.1
ALL_99	Cluster_2	0.005	0.989	0.006	4.9
ALL_98	Cluster_2	0.003	0.994	0.003	4.72
ALL_100	Cluster_3	0.035	0.011	0.955	4.62
ALL_1	Cluster_3	0.055	0.04	0.905	4.56

### 4.7 Implications for breeding and conservation of onion germplasm

Overall, the results show that the SRGB onion collection contains nuclear genetic diversity organized into distinct clusters, but phenotypic differentiation and breeding value are only partially explained by this structure. Nevertheless, several limitations should be acknowledged. First, only four individuals per accession were genotyped, so rare within-accession alleles or less frequent cytoplasmic profiles may not have been fully captured. Second, the phenotypic evaluation was conducted as a germplasm characterization trial rather than as a replicated field experiment. Environmental variance and plot-level experimental error could therefore not be estimated separately. The five bulbs evaluated per accession were treated as within-accession subsamples, not as field replicates. All accessions were grown in the same field, season, and management conditions to reduce environmental variation. However, additional multi-location or multi-year trials would be needed to test genotype × environment interactions, particularly for compositional traits such as dry matter content and soluble solids (Kazimierczak et al., 2021). Third, although the SSR and ILP marker set provided strong discriminatory power, genome-wide SNP approaches would likely offer finer resolution of population structure and trait-associated variation (Delvento et al., 2022). Despite these limitations, the SRGB onion collection can be regarded as a genetically diverse and internally variable germplasm resource. Adaptive potential and breeding value appear to be broadly distributed within this material. Nuclear differentiation likely reflects historical divergence and selection related to bulb architecture, but superior agronomic performance is not confined to a single genetic background. In the SRGB gene bank collection, these results underscore the importance of maintaining both within-accession diversity and differentiation among the major genetic clusters during regeneration and long-term conservation. For gene bank management, this means that regeneration strategies should preserve internal variability within individual landraces as well as the broader genetic structure of the collection. For breeding, the three nuclear clusters identified in this study provide a practical framework for exploiting genetic divergence while preserving the adaptive variability within each landrace. In this way, the SRGB collection represents not only a repository of allelic diversity but also a structured and functionally relevant resource for future onion improvement. Because breeding value is distributed across the structured diversity of the collection, this resource should be evaluated at the accession level for effective use. Cluster assignment alone is therefore not sufficient for selecting material for practical use.

## 5 Conclusion

The results present an integrated view of nuclear diversity, cytoplasmic polymorphism, population structure, and phenotypic differentiation in the SRGB onion collection. Nuclear SSR and ILP markers revealed substantial genetic diversity. They also identified three main nuclear genetic clusters, indicating a clear primary subdivision within the collection. Most nuclear variation occurred within accessions, confirming the heterogeneous nature of this open-pollinated germplasm. Cytoplasmic marker analysis detected widespread S-associated mitochondrial polymorphism. However, cytoplasmic profiles showed no clear correspondence with nuclear genetic structure. Phenotypic analyses showed measurable differences among nuclear clusters. These differences were mainly related to bulb size and morphology. Compositional traits, such as dry matter content and total soluble solids, showed weaker structuring. However, ancestry-based regressions and Selection Index results indicate that high-ranking breeding-oriented material is distributed across all genetic clusters rather than concentrated in a single cluster. This shows that genetic structure is useful for describing broad diversity, but it is not sufficient on its own for selecting the most promising accessions. Overall, the SRGB onion collection represents a genetically structured germplasm resource. It also shows broad phenotypic and genetic variation. This variation provides useful breeding-oriented potential. Effective conservation will therefore require maintaining both within-accession diversity and differentiation among major genetic clusters. At the same time, the identified structure provides a useful framework for breeding strategies. This structure may help exploit genetic divergence while preserving diversity within SRGB onion germplasm. These findings support accession-level evaluation as a practical approach for gene bank management and onion breeding.

## Data Availability

The datasets supporting the findings of this study are included in the article and its **Supplementary Material**. Further inquiries can be directed to the corresponding author.
