# Supplementary material for: Integrated molecular and phenotypic analysis of onion (Allium cepa L.) germplasm reveals limited correspondence between genetic structure and phenotypic traits
Source: Front Plant Sci. 2026 Jun 3;17:1849494. doi: 10.3389/fpls.2026.1849494 (PMC13272429; doi:10.3389/fpls.2026.1849494)
Supplement: Supplementary file 1 [file DataSheet1.docx]

Integrated Molecular and Phenotypic Analysis of Onion (*Allium cepa* L.) Germplasm Reveals Limited Correspondence between Genetic Structure and Phenotypic Traits

**Supplementary Table S1.** List of onion accessions from the Slovenian Plant Gene Bank (SRGB) and reference cultivars included in this study.

| **Accession ID** | **SRGB code** | **Taxon** | **Accession name** | **Country of origin** | **Collection origin** | **Latitude (°N)** | **Longitude (°E)** | **Accession status** |
| --- | --- | --- | --- | --- | --- | --- | --- | --- |
| ALL-1 | SRGB 4363 | *Allium cepa* | Ptujska rdeča | Slovenia | Stojnci | 46.38121419 | 15.97220615 | Traditional cultivar / landrace |
| ALL-2 | SRGB 4364 | *Allium cepa* | Ptujska rdeča | Slovenia | Stojnci | 46.38059991 | 15.97743318 | Traditional cultivar / landrace |
| ALL-4 | SRGB 4366 | *Allium cepa* | Ptujska rdeča | Slovenia | Stojnci | 46.38178401 | 15.98149964 | Traditional cultivar / landrace |
| ALL-5 | SRGB 4367 | *Allium cepa* | Ptujska rdeča | Slovenia | Stojnci | 46.3799305 | 15.97800914 | Traditional cultivar / landrace |
| ALL-7 | SRGB 4368 | *Allium cepa* | Ptujska rdeča | Slovenia | Stojnci | 46.38143849 | 15.98024711 | Traditional cultivar / landrace |
| ALL-8 | SRGB 4369 | *Allium cepa* | Ptujska rdeča | Slovenia | Stojnci | 46.3818919 | 15.97166424 | Traditional cultivar / landrace |
| ALL-9 | SRGB 4370 | *Allium cepa* | Ptujska rdeča | Slovenia | Stojnci | 46.37627551 | 15.97970542 | Traditional cultivar / landrace |
| ALL-10 | SRGB 4371 | *Allium cepa* | Ptujska rdeča | Slovenia | Stojnci | 46.3805566 | 15.97205291 | Traditional cultivar / landrace |
| ALL-11 | SRGB 4372 | *Allium cepa* | Ptujska rdeča | Slovenia | Stojnci | 46.3822486 | 15.98395354 | Traditional cultivar / landrace |
| ALL-12 | SRGB 4373 | *Allium cepa* | Ptujska rdeča | Slovenia | Stojnci | 46.37224392 | 15.97487204 | Traditional cultivar / landrace |
| ALL-13 | SRGB 4374 | *Allium cepa* | Ptujska rdeča | Slovenia | Stojnci | 46.38232599 | 15.96950645 | Traditional cultivar / landrace |
| ALL-14 | SRGB 4375 | *Allium cepa* | Ptujska rdeča | Slovenia | Stojnci | 46.38023421 | 15.97644757 | Traditional cultivar / landrace |
| ALL-16 | SRGB 3939 | *Allium cepa* | Ptujska rdeča | Slovenia | Stojnci | 46.3809886 | 15.97940914 | Traditional cultivar / landrace |
| ALL-17 | SRGB 3940 | *Allium cepa* | Ptujska rdeča | Slovenia | Stojnci | 46.3824618 | 15.98454618 | Traditional cultivar / landrace |
| ALL-18 | SRGB 4376 | *Allium cepa* | Ptujska rdeča | Slovenia | Stojnci | 46.3811686 | 15.97999808 | Traditional cultivar / landrace |
| ALL-21 | SRGB 4377 | *Allium cepa* | Ptujska rdeča | Slovenia | Stojnci | 46.3790896 | 15.97361892 | Traditional cultivar / landrace |
| ALL-22 | SRGB 3941 | *Allium cepa* | Ptujska rdeča | Slovenia | Stojnci | 46.37668852 | 15.98188565 | Traditional cultivar / landrace |
| ALL-27 | SRGB 3942 | *Allium cepa* | Ptujska rdeča | Slovenia | Bukovci | 46.38815298 | 15.9574564 | Traditional cultivar / landrace |
| ALL-37 | SRGB 3943 | *Allium cepa* | Ptujska rdeča | Slovenia | Bukovci | 46.38082431 | 15.96733993 | Traditional cultivar / landrace |
| ALL-39 | SRGB 5770 | *Allium cepa* | – | Slovenia | Bukovci | 46.37594931 | 15.95261462 | Traditional cultivar / landrace |
| ALL-48 | SRGB 5771 | *Allium cepa* | – | Slovenia | Moretinci | 46.3829764 | 15.98778892 | Traditional cultivar / landrace |
| ALL-50 | SRGB 3944 | *Allium cepa* | Ptujska rdeča | Slovenia | Dornava | 46.43228163 | 15.94934338 | Traditional cultivar / landrace |
| ALL-53 | SRGB 4379 | *Allium cepa* | – | Slovenia | Višnja Gora | 45.95454519 | 14.74423352 | Traditional cultivar / landrace |
| ALL-54 | SRGB 4380 | *Allium cepa* | – | Slovenia | Dobrova | 46.03958056 | 14.39771834 | Traditional cultivar / landrace |
| ALL-55 | SRGB 3945 | *Allium cepa* | – | Slovenia | Velika Loka | 45.95189641 | 14.92924488 | Traditional cultivar / landrace |
| ALL-57 | SRGB 4381 | *Allium cepa* | – | Slovenia | Trebnje | 45.89913948 | 14.94145217 | Traditional cultivar / landrace |
| ALL-58 | SRGB 4382 | *Allium cepa* | – | Slovenia | Velika Loka | 45.93658426 | 14.94927057 | Traditional cultivar / landrace |
| ALL-59 | SRGB 4383 | *Allium cepa* | – | Slovenia | Velika Loka | 45.95212206 | 14.93002644 | Traditional cultivar / landrace |
| ALL-62 | SRGB 3946 | *Allium cepa* | Ptujska rdeča | Slovenia | Zgornja Polskava | 46.4235286 | 15.61647753 | Traditional cultivar / landrace |
| ALL-64 | SRGB 5772 | *Allium cepa* | – | Slovenia | Stojnci | 46.37309549 | 15.97473025 | Traditional cultivar / landrace |
| ALL-66 | SRGB 5773 | *Allium cepa* | – | Slovenia | Bukovci | 46.38895878 | 15.95099125 | Traditional cultivar / landrace |
| ALL-70 | SRGB 3948 | *Allium cepa* | Belokranjka | Slovenia | Gradac | 45.5721401 | 15.29068717 | Traditional cultivar / landrace |
| ALL-71 | SRGB 3949 | *Allium cepa* | Belokranjka | Slovenia | Gradac | 45.5699058 | 15.2884262 | Traditional cultivar / landrace |
| ALL-72 | SRGB 3950 | *Allium cepa* | Ptujska rdeča | Slovenia | Markovci | 46.38438449 | 15.93682811 | Traditional cultivar / landrace |
| ALL-75 | SRGB 3951 | *Allium cepa* | Ptujska rdeča | Slovenia | Gorišnica | 46.39346276 | 16.0100653 | Traditional cultivar / landrace |
| ALL-76 | SRGB 4384 | *Allium cepa* | – | Slovenia | Velika Loka | 45.95215935 | 14.92999425 | Traditional cultivar / landrace |
| ALL-79 | SRGB 4385 | *Allium cepa* | – | Slovenia | Velika Loka | 45.93659172 | 14.94930275 | Traditional cultivar / landrace |
| ALL-80 | SRGB 5775 | *Allium cepa* | – | Slovenia | Velika Loka | 45.94928253 | 14.92404224 | Traditional cultivar / landrace |
| ALL-84 | SRGB 5776 | *Allium cepa* | – | Slovenia | Brezovica | 46.02224342 | 14.37577468 | Traditional cultivar / landrace |
| ALL-85 | SRGB 4386 | *Allium cepa* | – | Slovenia | Trebnje | 45.8982435 | 14.94158091 | Traditional cultivar / landrace |
| ALL-86 | SRGB 5777 | *Allium cepa* | – | Slovenia | Velika Loka | 45.95243532 | 14.92978515 | Traditional cultivar / landrace |
| ALL-87 | SRGB 4387 | *Allium cepa* | – | Slovenia | Velika Loka | 45.95182928 | 14.92916978 | Traditional cultivar / landrace |
| ALL-88 | SRGB 3952 | *Allium cepa* | Ptujska rdeča | Slovenia | Dornava | 46.43512922 | 15.95217162 | Traditional cultivar / landrace |
| ALL-89 | SRGB 3953 | *Allium cepa* | Ptujska rdeča | Slovenia | Dornava | 46.43899674 | 15.95242046 | Traditional cultivar / landrace |
| ALL-90 | SRGB 3954 | *Allium cepa* | Ptujska rdeča | Slovenia | Dornava | 46.43606805 | 15.95285374 | Traditional cultivar / landrace |
| ALL-92 | SRGB 3955 | *Allium cepa* | Ptujska rdeča | Slovenia | Dornava | 46.43739082 | 15.95413388 | Traditional cultivar / landrace |
| ALL-94 | SRGB 3957 | *Allium cepa* | Ptujska rdeča | Slovenia | Dornava | 46.43302814 | 15.95844588 | Traditional cultivar / landrace |
| ALL-95 | SRGB 3958 | *Allium cepa* | Ptujska rdeča | Slovenia | Dornava | 46.43543553 | 15.95392852 | Traditional cultivar / landrace |
| ALL-96 | SRGB 5779 | *Allium cepa* | – | Slovenia | Dornava | 46.43772043 | 15.95535541 | Traditional cultivar / landrace |
| ALL-97 | SRGB 3959 | *Allium cepa* | Ptujska rdeča | Slovenia | Dornava | 46.43760346 | 15.95573311 | Traditional cultivar / landrace |
| ALL-98 | SRGB 3960 | *Allium cepa* | Ptujska rdeča | Slovenia | Dornava | 46.43920901 | 15.95147457 | Traditional cultivar / landrace |
| ALL-99 | SRGB 3961 | *Allium cepa* | Ptujska rdeča | Slovenia | Dornava | 46.4368103 | 15.95840631 | Traditional cultivar / landrace |
| ALL-100 | SRGB 3962 | *Allium cepa* | Ptujska rdeča | Slovenia | Dornava | 46.43564369 | 15.95528882 | Traditional cultivar / landrace |
| ALL-101 | SRGB 3963 | *Allium cepa* | Belokranjka, long yellow | Slovenia | Gradac | 45.57581636 | 15.29258289 | Traditional cultivar / landrace |
| ALL-102 | SRGB 3964 | *Allium cepa* | Belokranjka | Slovenia | Gradac | 45.57353682 | 15.29301264 | Traditional cultivar / landrace |
| ALL-103 | SRGB 3965 | *Allium cepa* | Belokranjka | Slovenia | Gradac | 45.57127749 | 15.28992487 | Traditional cultivar / landrace |
| ALL-104 | SRGB 3966 | *Allium cepa* | Belokranjka | Slovenia | Gradac | 45.57456088 | 15.29370484 | Traditional cultivar / landrace |
| ALL-106 | SRGB 3970 | *Allium cepa* | Raška | Slovenia | Krško | 45.93109907 | 15.37776882 | Traditional cultivar / landrace |
| ALL-107 | SRGB 3971 | *Allium cepa* | – | Italia | Elba | 42.79568033 | 10.3378392 | Traditional cultivar / landrace |

| Reference cultivars | | | |
| --- | --- | --- | --- |
| Reference cultivars | **Type** | **Scientific name** | **Supplier/source** |
| Talon | Hybrid cultivar | *Allium cepa* | Agrocasol Plus |
| Holandska rumena | Commercial cultivar | *Allium cepa* | Semenarna Ljubljana |
| Ptujska rdeča | Commercial cultivar | *Allium cepa* | Semenarna Ljubljana |

Accessions ALL-50 and ALL-75 were excluded from the phenotypic evaluation because they did not germinate.

**Supplementary Table S2.** Markers used for onion genotyping. A total of 26 markers were used, including five cytoplasmic PCR markers, 15 SSR markers, and six ILP markers. Primer sequences, Marker tpye, fluorescent label, PCR protocols or annealing temperature, and literature reference are shown.

| **Marker** | **Forward primer (5′–3′)** | **Reverse primer (5′–3′)** | **Marker type** | **Fluorescent label** | **PCR protocol / annealing temperature** | **Reference** |
| --- | --- | --- | --- | --- | --- | --- |
| orfA501 | ATGGCTCGCCTTGAAAGAGAGC | CCAAGCATTTGGCGCTGAC | Cytoplasmic PCR | – | Ta = 60°C | Engelke et al., 2003 |
| 5′cob_S | GTCCAGTTCCTATAGAACCTATCACT | CTTTTCTATGGTGACAACTCCTCTT | Cytoplasmic PCR | – | Ta = 53°C | Engelke et al., 2003 |
| 5′cob_N | TCTAGATGTCGCATCAGTGGAATCC | CTTTTCTATGGTGACAACTCCTCTT | Cytoplasmic PCR | – | Ta = 53°C | Engelke et al., 2003 |
| OPT | CCTTGGAAAGGCGCAACTAAAGATTTGA | TGTGGCCCAATAATACAAACAAGCAGGA | Cytoplasmic PCR | – | Ta = 60°C | Bang et al., 2011 |
| PSAO | CCTCATGCTTGCTTGGTCTT | AAGCGTGATCGATTGTAGGTCCTTT | Cytoplasmic PCR | – | Touchdown Ta = 65°C to 57°C, followed by Ta = 57°C | Bang et al., 2011 |
| AMS08 | GCCACGATGTTGAGATTTCG | CCCGAATATCCCACCAGTTC | SSR | FAM | Modified Piquemal | Fischer and Bachmann, 2000 |
| AMS23 | GCTGTTCACTGGTCTATCTGG | ATTCGGTGCTGATTTTCG | SSR | NED | Modified Piquemal | Fischer and Bachmann, 2000 |
| AMS25 | GAGGGCAGTGTTAGCATTCC | GCAACCTTTCCCCGAGAG | SSR | HEX | Piquemal | Fischer and Bachmann, 2000 |
| AMS26 | ATCTAATCAAAGCATAGTTG | TTGTCCAAGTAGTTGTGA | SSR | FAM | Modified Piquemal | Fischer and Bachmann, 2000 |
| AMS12 | AATGTTGCTTTCTTTAGATGTTG | TGCAAAATTACAAGCAAACTG | SSR | NED | Modified Piquemal | Fischer and Bachmann, 2000 |
| AMS17 | AGTGGACTCAAGGCAGATG | ATCACCATTCACCGTTTACT | SSR | HEX | Piquemal | Fischer and Bachmann, 2000 |
| AMS21 | GGTTGTTTCCACTACACTTGAG | CGTCCTTGGTATTCTTGTGC | SSR | FAM | Piquemal | Fischer and Bachmann, 2000 |
| AcILP58 | CATGTCGCAAAAGTGTTTGG | CCAATCTCATTACCCAGTCA | ILP | NED | Piquemal | Jayaswall et al., 2019 |
| AcILP48 | ATTGAATCCACCTGGCAAAAG | CCAGCTGAGAGGGTTGTAGG | ILP | HEX | Modified Piquemal | Jayaswall et al., 2019 |
| AcILP47 | ATTCAATGATGCCAGAGTGC | TGCAATTTTCTCCCATTGTTC | ILP | FAM | Modified Piquemal | Jayaswall et al., 2019 |
| AcILP112 | GCTTTGCAAGCATTACACCA | AGTCCATCTCCCCACATCT | ILP | NED | Piquemal | Jayaswall et al., 2019 |
| AcILP93 | GATTCGCGTTGGACATTTCT | GCTCTGGAGCCATCCATCTA | ILP | HEX | SSR-tail | Jayaswall et al., 2019 |
| AcILP103 | GCAGATCCATGGCCTAGTATT | AACCGAGTTAGGACAGCAGAA | ILP | FAM | SSR-tail | Jayaswall et al., 2019 |
| ACM033 | CCTTCTCCCCATTCTCTTCC | ATCATCGTCCTCGTCCTAC | SSR | NED | Modified Piquemal | McCallum et al., 2008 |
| ACM038 | ATGCCAGACTACGACAACGA | ACGCCTACCAACCTTCAATG | SSR | HEX | Piquemal | McCallum et al., 2008 |
| ACM045 | AAAACGAAGCAACAAACAAA | CGACGAAGGTCATAAGTAGGC | SSR | FAM | Piquemal | McCallum et al., 2008 |
| ACM054 | GAGTGAGAGGGGAAATGGAA | AAAGATGGTTTGTTGGTGGC | SSR | NED | Piquemal | McCallum et al., 2008 |
| ACM058 | GGAGTCACACAACAGAAACACAA | AAGAAGGAATAGAGATGTAGCCGA | SSR | HEX | SSR-tail | McCallum et al., 2008 |
| ACM077 | AAATTATGGGCCACCTCCTC | CAAGATTGTCGACTCCCCAT | SSR | FAM | SSR-tail | McCallum et al., 2008 |
| ACM094 | GATGATGGCAAGACACAGA | AAAACGGCTTAGGAATTTAAACG | SSR | NED | Piquemal | McCallum et al., 2008 |
| ACM147 | CACTTTCCCGTCTAATCGACA | TTCCCACAATCAAAACACCA | SSR | HEX | Piquemal | McCallum et al., 2008 |

Ta, annealing temperature. Piquemal protocol: 15 cycles at 49.5°C + 0.7°C per cycle, followed by 23 cycles at 53°C. Modified Piquemal protocol: 30 cycles at 49.5°C + 0.5°C per cycle, followed by 30 cycles at 49.5°C. SSR-tail protocol: 10 touchdown cycles from 56°C to 55°C, followed by 30 cycles at 55°C.

**Supplementary Table S3A.** Ordinal morphological descriptors used for onion phenotypic evaluation. Traits were scored using discrete scales adapted from UPOV (2008) and CPVO (2009).

| **No.** | **Trait** | **Code** | **Description** | **Scale** |
| --- | --- | --- | --- | --- |
| 1 | Foliage attitude | FA | Plant foliage orientation | 1 erect; 2 erect–semi-erect; 3 semi-erect; 4 semi-erect–horizontal; 5 horizontal |
| 2 | Foliage waxiness | FW | Leaf wax layer intensity | 1 absent/very weak; 3 weak; 5 medium; 7 strong; 9 very strong |
| 3 | Foliage: intensity of green color | FGI | Leaf color intensity | 1 very light; 3 light; 5 medium; 7 dark |
| 4 | Bulb size | BS | Overall bulb size class | 3 small; 5 medium; 7 large |
| 5 | Bulb_Bulblet: intensity of base color of dry skin | BDSI_BB | Intensity of bulb base color | 3 light; 5 medium; 7 dark |

**Supplementary Table S3B.** Categorical morphological descriptors used for onion phenotypic evaluation. Variables were recorded as binary values (presence = 1, absence = 0).

|  | **Descriptor group** | **Variable** | **Category** |
| --- | --- | --- | --- |
| 1 | Position of maximum diameter | PST_E | Towards stem end |
| 2 |  | PST_M | At middle |
| 3 |  | PRT_E | Towards root end |
| 4 | Bulb shape (longitudinal section) | SL1_EL | Elliptic |
| 5 |  | SL2_MO | Medium ovate |
| 6 |  | SL3_BE | Broad elliptic |
| 7 |  | SL4_CI | Circular |
| 8 |  | SL5_BO | Broad ovate |
| 9 |  | SL6_BOB | Broad obovate |
| 10 |  | SL7_RH | Rhombic |
| 11 |  | SL8_TME | Transverse medium elliptic |
| 12 |  | SL9_TNE | Transverse narrow elliptic |
| 13 | Shape of stem end | SS1_DEP | Depressed |
| 14 |  | SS2_FL | Flat |
| 15 |  | SS3_RO | Slightly raised / rounded |
| 16 |  | SS4_WT | Weakly tapered |
| 17 |  | SS5_ST | Strongly tapered |
| 18 |  | SS6_PO | Pointed |
| 19 | Shape of root end | SR1_DEP | Depressed |
| 20 |  | SR2_FL | Flat |
| 21 |  | SR3_RO | Round |
| 22 |  | SR4_WT | Weakly tapered |
| 23 |  | SR5_ST | Strongly tapered |
| 24 | Base color of dry skin | BDS1_WH | White |
| 25 |  | BDS2_GR | Grey |
| 26 |  | BDS3_GN | Green |
| 27 |  | BDS4_YE | Yellow |
| 28 |  | BDS5_BR | Brown |
| 29 |  | BDS6_PI | Pink |
| 30 |  | BDS7_RD | Red |
| 31 |  | BDS8_PU | Purple |
| 32 | Epidermis color of fleshy scales | EC1_AB | Absent |
| 33 |  | EC2_GN | Greenish |
| 34 |  | EC3_RD | Reddish |

**Supplementary Table S3C.** Continuous quantitative traits measured during onion phenotypic evaluation.

|  | **Trait** | **Code** | **Unit** |
| --- | --- | --- | --- |
| 1 | Bulb weight | BW | g |
| 2 | Bulb height | BH | mm |
| 3 | Bulb diameter | BD | mm |
| 4 | Neck width | BNW | mm |
| 5 | Height/diameter ratio | HDR | – |
| 6 | Total soluble solids | TSS | °Brix |
| 7 | Number of fleshy scale leaves | NSL | count |
| 8 | Dry matter content | DMC | % |

**Supplementary Table S4.** Assignment of onion genotypes (Line) to STRUCTURE genetic clusters (K = 3).

| Cluster 3 | | Cluster 1 | | Cluster 2 | |
| --- | --- | --- | --- | --- | --- |
| ID | **Line** | **ID** | **Line** | **ID** | **Line** |
| 1 | ALL_1 | 24 | ALL_48 | 46 | ALL_86 |
| 2 | ALL_10 | 25 | ALL_5 | 47 | ALL_87 |
| 3 | ALL_100 | 26 | ALL_53 | 48 | ALL_88 |
| 4 | ALL_101 | 27 | ALL_54 | 49 | ALL_89 |
| 5 | ALL_102 | 28 | ALL_55 | 50 | ALL_9 |
| 6 | ALL_103 | 29 | ALL_57 | 51 | ALL_90 |
| 7 | ALL_104 | 30 | ALL_58 | 52 | ALL_92 |
| 8 | ALL_106 | 31 | ALL_59 | 53 | ALL_94 |
| 9 | ALL_107 | 32 | ALL_62 | 54 | ALL_95 |
| 10 | ALL_11 | 33 | ALL_64 | 55 | ALL_96 |
| 11 | ALL_12 | 34 | ALL_66 | 56 | ALL_97 |
| 12 | ALL_13 | 35 | ALL_7 | 57 | ALL_98 |
| 13 | ALL_14 | 36 | ALL_70 | 58 | ALL_99 |
| 14 | ALL_16 | 37 | ALL_71 |  |  |
| 15 | ALL_17 | 38 | ALL_72 |  |  |
| 16 | ALL_18 | 39 | ALL_76 |  |  |
| 17 | ALL_2 | 40 | ALL_77 |  |  |
| 18 | ALL_21 | 41 | ALL_79 |  |  |
| 19 | ALL_22 | 42 | ALL_8 |  |  |
| 20 | ALL_27 | 43 | ALL_80 |  |  |
| 21 | ALL_37 | 44 | ALL_84 |  |  |
| 22 | ALL_39 | 45 | ALL_85 |  |  |
| 23 | ALL_4 |  |  |  |  |

**Supplementary Table S5.** Multiple regression analysis of phenotypic traits as a function of STRUCTURE ancestry coefficients (Trait ~ Q1 + Q2). The table reports regression coefficients (β ± SE), 95% confidence intervals, nominal p-values, FDR-adjusted p-values, and model summary statistics (R², adjusted R², model p-value, and FDR-adjusted model p-value).

| **Trait** | **Term** | **β** | **SE** | **CI_low** | **CI_high** | **p-value** | **FDR_p** | **R²** | **Adj_R²** | **Model_p** | **Model_FDR** |
| --- | --- | --- | --- | --- | --- | --- | --- | --- | --- | --- | --- |
| Bulb weight | Q1 | -11.35 | 8.35 | -27.73 | 5.02 | 0.18 | 0.35 | 0.13 | 0.10 | 0.02 | 0.17 |
| Bulb weight | Q2 | 14.67 | 8.68 | -2.34 | 31.67 | 0.10 | 0.23 | 0.13 | 0.10 | 0.02 | 0.17 |
| Bulb diameter | Q1 | 0.03 | 2.18 | -4.23 | 4.30 | 0.99 | 0.99 | 0.21 | 0.18 | 0.11 | 0.24 |
| Bulb diameter | Q2 | 7.75 | 2.26 | 3.32 | 12.18 | 0.00 | 0.01 | 0.21 | 0.18 | 0.11 | 0.24 |
| Bulb height | Q1 | -8.54 | 5.00 | -18.34 | 1.25 | 0.09 | 0.33 | 0.05 | 0.02 | 0.16 | 0.24 |
| Bulb height | Q2 | -5.22 | 5.19 | -15.40 | 4.96 | 0.32 | 0.45 | 0.05 | 0.02 | 0.16 | 0.24 |
| Neck width | Q1 | -0.96 | 0.74 | -2.41 | 0.49 | 0.20 | 0.35 | 0.06 | 0.03 | 0.08 | 0.24 |
| Neck width | Q2 | 0.57 | 0.77 | -0.94 | 2.08 | 0.46 | 0.54 | 0.06 | 0.03 | 0.08 | 0.24 |
| Height/diameter ratio | Q1 | -0.16 | 0.09 | -0.35 | 0.02 | 0.09 | 0.33 | 0.09 | 0.05 | 0.36 | 0.36 |
| Height/diameter ratio | Q2 | -0.21 | 0.10 | -0.40 | -0.01 | 0.04 | 0.14 | 0.09 | 0.05 | 0.36 | 0.36 |
| Total soluble solids | Q1 | 0.38 | 0.36 | -0.32 | 1.09 | 0.29 | 0.41 | 0.02 | -0.01 | 0.29 | 0.34 |
| Total soluble solids | Q2 | 0.10 | 0.37 | -0.64 | 0.83 | 0.80 | 0.80 | 0.02 | -0.01 | 0.29 | 0.34 |
| Dry matter content | Q1 | 0.38 | 0.47 | -0.53 | 1.30 | 0.41 | 0.48 | 0.05 | 0.02 | 0.17 | 0.24 |
| Dry matter content | Q2 | -0.49 | 0.48 | -1.44 | 0.46 | 0.31 | 0.45 | 0.05 | 0.02 | 0.17 | 0.24 |

**Supplementary Table S6. O**nion accessions ranked by Selection Index (SI), including STRUCTURE-derived cluster assignment (K = 3) and ancestry proportions (Q1, Q2, Q3).

| Accession | Selection Index | Rank SI | Cluster | Q1 | Q2 | Q3 |
| --- | --- | --- | --- | --- | --- | --- |
| ALL_89 | 6.44 | 1 | Cluster_2 | 0.004 | 0.992 | 0.004 |
| ALL_106 | 5.93 | 2 | Cluster_3 | 0.031 | 0.006 | 0.964 |
| ALL_87 | 5.48 | 3 | Cluster_2 | 0.005 | 0.891 | 0.104 |
| ALL_95 | 5.18 | 4 | Cluster_2 | 0.005 | 0.989 | 0.006 |
| ALL_17 | 5.15 | 5 | Cluster_3 | 0.004 | 0.003 | 0.993 |
| ALL_70 | 5.1 | 6 | Cluster_1 | 0.838 | 0.004 | 0.158 |
| ALL_99 | 4.9 | 7 | Cluster_2 | 0.005 | 0.989 | 0.006 |
| ALL_98 | 4.72 | 8 | Cluster_2 | 0.003 | 0.994 | 0.003 |
| ALL_100 | 4.62 | 9 | Cluster_3 | 0.035 | 0.011 | 0.955 |
| ALL_1 | 4.56 | 10 | Cluster_3 | 0.055 | 0.04 | 0.905 |
| ALL_79 | 4.49 | 11 | Cluster_1 | 0.918 | 0.055 | 0.027 |
| ALL_97 | 4.48 | 12 | Cluster_2 | 0.006 | 0.991 | 0.003 |
| ALL_76 | 4.47 | 13 | Cluster_1 | 0.914 | 0.015 | 0.072 |
| ALL_103 | 4.44 | 14 | Cluster_3 | 0.113 | 0.003 | 0.884 |
| ALL_96 | 4.24 | 15 | Cluster_2 | 0.014 | 0.981 | 0.006 |
| ALL_71 | 4.23 | 16 | Cluster_1 | 0.994 | 0.004 | 0.002 |
| ALL_107 | 4.15 | 17 | Cluster_3 | 0.028 | 0.004 | 0.968 |
| ALL_86 | 4.06 | 18 | Cluster_2 | 0.013 | 0.976 | 0.011 |
| ALL_104 | 3.98 | 19 | Cluster_3 | 0.149 | 0.008 | 0.843 |
| ALL_37 | 3.79 | 20 | Cluster_3 | 0.014 | 0.009 | 0.976 |
| ALL_27 | 3.76 | 21 | Cluster_3 | 0.078 | 0.012 | 0.909 |
| ALL_7 | 3.74 | 22 | Cluster_1 | 0.994 | 0.004 | 0.002 |
| ALL_22 | 3.66 | 23 | Cluster_3 | 0.011 | 0.011 | 0.977 |
| ALL_102 | 3.62 | 24 | Cluster_3 | 0.205 | 0.007 | 0.788 |
| ALL_53 | 3.61 | 25 | Cluster_1 | 0.908 | 0.005 | 0.087 |
| ALL_5 | 3.6 | 26 | Cluster_1 | 0.712 | 0.152 | 0.136 |
| ALL_62 | 3.58 | 27 | Cluster_1 | 0.99 | 0.005 | 0.005 |
| ALL_8 | 3.54 | 28 | Cluster_1 | 0.754 | 0.004 | 0.243 |
| ALL_85 | 3.54 | 29 | Cluster_1 | 0.497 | 0.452 | 0.051 |
| ALL_92 | 3.44 | 30 | Cluster_2 | 0.033 | 0.96 | 0.007 |
| ALL_10 | 3.34 | 31 | Cluster_3 | 0.055 | 0.009 | 0.936 |
| ALL_88 | 3.34 | 32 | Cluster_2 | 0.005 | 0.987 | 0.008 |
| ALL_72 | 3.33 | 33 | Cluster_1 | 0.879 | 0.005 | 0.117 |
| ALL_84 | 3.32 | 34 | Cluster_1 | 0.801 | 0.005 | 0.194 |
| ALL_66 | 3.22 | 35 | Cluster_1 | 0.941 | 0.004 | 0.056 |
| ALL_77 | 3.2 | 36 | Cluster_1 | 0.991 | 0.003 | 0.006 |
| ALL_39 | 3.19 | 37 | Cluster_3 | 0.101 | 0.008 | 0.891 |
| ALL_59 | 3.1 | 38 | Cluster_1 | 0.991 | 0.003 | 0.006 |
| ALL_4 | 3.09 | 39 | Cluster_3 | 0.193 | 0.015 | 0.792 |
| ALL_94 | 2.99 | 40 | Cluster_2 | 0.031 | 0.965 | 0.004 |
| ALL_80 | 2.96 | 41 | Cluster_1 | 0.93 | 0.014 | 0.055 |
| ALL_16 | 2.92 | 42 | Cluster_3 | 0.032 | 0.004 | 0.963 |
| ALL_54 | 2.9 | 43 | Cluster_1 | 0.964 | 0.005 | 0.031 |
| ALL_58 | 2.78 | 44 | Cluster_1 | 0.819 | 0.013 | 0.169 |
| ALL_2 | 2.62 | 45 | Cluster_3 | 0.027 | 0.003 | 0.97 |
| ALL_90 | 2.58 | 46 | Cluster_2 | 0.003 | 0.994 | 0.003 |
| ALL_18 | 2.48 | 47 | Cluster_3 | 0.002 | 0.002 | 0.996 |
| ALL_101 | 2.48 | 48 | Cluster_3 | 0.015 | 0.065 | 0.92 |
| ALL_9 | 2.45 | 49 | Cluster_2 | 0.006 | 0.992 | 0.002 |
| ALL_57 | 2.26 | 50 | Cluster_1 | 0.929 | 0.009 | 0.063 |
| ALL_13 | 2.17 | 51 | Cluster_3 | 0.003 | 0.008 | 0.99 |
| ALL_12 | 1.9 | 52 | Cluster_3 | 0.014 | 0.006 | 0.98 |
| ALL_64 | 1.83 | 53 | Cluster_1 | 0.993 | 0.004 | 0.004 |
| ALL_55 | 1.62 | 54 | Cluster_1 | 0.891 | 0.007 | 0.102 |
| ALL_11 | 1.52 | 55 | Cluster_3 | 0.061 | 0.009 | 0.93 |
| ALL_48 | 1.46 | 56 | Cluster_1 | 0.701 | 0.228 | 0.072 |
| ALL_14 | 1.35 | 57 | Cluster_3 | 0.003 | 0.002 | 0.995 |
| ALL_21 | 1 | 58 | Cluster_3 | 0.009 | 0.012 | 0.979 |

**Supplementary Table S7A.** One-way ANOVA testing differences in Selection Index (SI) among STRUCTURE-defined genetic clusters (K = 3).

| Source | Df | Sum Sq | Mean Sq | F value | Pr(>F) |
| --- | --- | --- | --- | --- | --- |
| Cluster | 2 | 8.12 | 4.06 | 3.25 | 0.046 |
| Residuals | 55 | 68.78 | 1.25 | – | – |

**Supplementary Table S7B.** Tukey’s HSD post-hoc pairwise comparisons of Selection Index (SI) among STRUCTURE-defined genetic clusters (K = 3).

| **Comparison** | **Diff** | **Lower** | **Upper** | **p adj** |
| --- | --- | --- | --- | --- |
| Cluster_2 – Cluster_1 | 0.90 | -0.03 | 1.85 | 0.06 |
| Cluster_3 – Cluster_1 | 0.02 | -0.77 | 0.83 | 0.99 |
| Cluster_3 – Cluster_2 | -0.88 | -1.81 | 0.05 | 0.06 |

**Supplementary Table S7C.** Linear regression analysis of Selection Index (SI) as a function of STRUCTURE ancestry coefficients (Q1 and Q2; Q3 implicit).

| Term | Estimate | SE | t value | p-value | CI_low | CI_high |
| --- | --- | --- | --- | --- | --- | --- |
| Intercept | 3.28 | 0.25 | 13.07 | <0.001 | 2.78 | 3.78 |
| Q1 | -0.02 | 0.39 | -0.06 | 0.95 | -0.81 | 0.76 |
| Q2 | 0.88 | 0.41 | 2.15 | 0.04 | 0.06 | 1.69 |

R² = 0.10; Adjusted R² = 0.06; F₂,₅₅ = 2.93; p = 0.062.


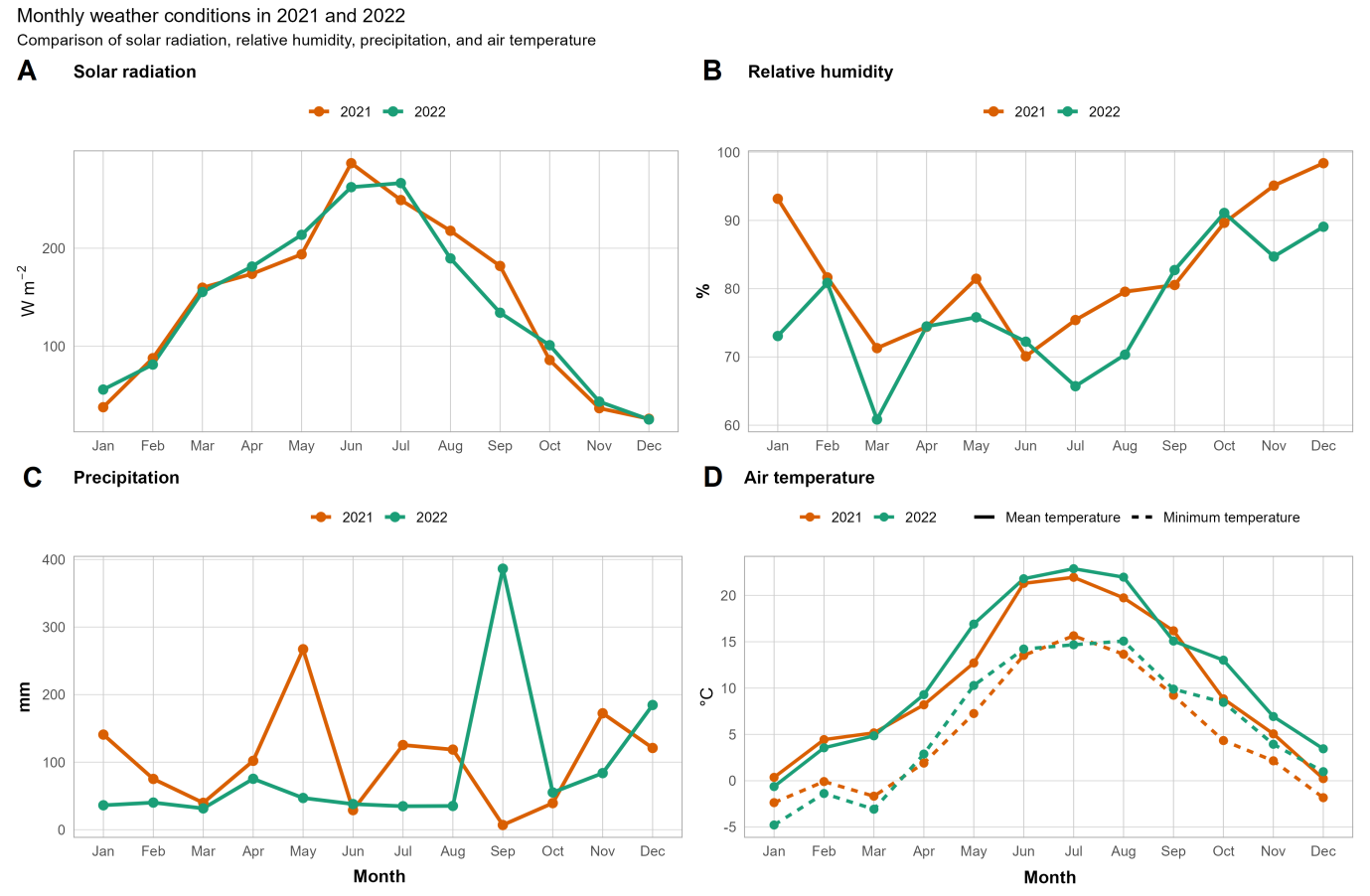


**Supplementary Figure S1.** Monthly weather conditions during 2021 and 2022 at the experimental site: (A) solar radiation, (B) relative humidity, (C) precipitation, and (D) air temperature. Solar radiation, relative humidity, and air temperature are shown as monthly means, while precipitation is presented as the monthly total. Panel D includes both mean and minimum air temperature.
